# MiR-26a-5p regulates proliferation, apoptosis, migration and invasion via inhibiting hydroxysteroid dehydrogenase like-2 in cervical cancer cell

**DOI:** 10.1186/s12885-022-09970-x

**Published:** 2022-08-10

**Authors:** Ming Li, Yubo Xiao, Minqi Liu, Qian Ning, Ziye Xiang, Xiang Zheng, Shengsong Tang, Zhongcheng Mo

**Affiliations:** 1 Hunan Province Key Laboratory for Antibody-Based Drug and Intelligent Delivery System, Hunan University of Medicine, Huaihua, 418000 Hunan China; 2 Guangxi Key Laboratory of Molecular Medicine in Liver Injury and Repair, Guilin Medical University, Guilin, 541001 Guangxi China; 3 Guangxi Province Postgraduate Co-Training Base for Cooperative Innovation in Basic Medicine (Guilin Medical University and Yueyang Women & Children’s Medical Center), Yueyang, 414000 China; 4 School of Medical Laboratory Science, Changsha Medical University, Changsha, 410000 Hunan China; 5 College of Bioscience and Biotechnology, Hunan Agricultural University, Changsha, 410128 Hunan China

**Keywords:** Cervical cancer, miR-26a-5p, HSDL2, Proliferation, Apoptosis

## Abstract

**Background:**

Evidences have indicated that miR-26a-5p regulates the malignant properties of various tumor cells. However, the influences of miR-26a-5p on proliferation, apoptosis and invasion are still vague in the cervical cancer (CC) cells.

**Methods:**

The miRNA microarray and real-time quantitative PCR (RT-qPCR) analysis were utilized to detect the expression of miR-26a-5p in the patients with CC. Kaplan–Meier plotter was performed to evaluate the overall survival (OS) of the patients with CC. The CCK-8, flow cytometry, transwell and wound healing analyses were respectively used to analyze proliferation, migration and invasion in the CC cells. RT-qPCR, western blot and IHC analysis were executed to measure the expression of hydroxysteroid dehydrogenase like-2 (HSDL2) in the patients with CC. Bioinformatics and luciferase reporter assay were carried out to verify the relationship of miR-26a-5p and HSDL2.

**Results:**

The expression of miR-26a-5p was downregulated and low expression of miR-26a-5p indicated a poor OS in patients with CC. Overexpression of miR-26a-5p significantly inhibited proliferation, migration and invasion, accelerated apoptosis in the Hela and C33A cells. The expression of HSDL2 was upregulated, and negatively correlated with miR-26a-5p in the patients with CC. HSDL2 was directly targeted by miR-26a-5p and rescue experiments displayed that HSDL2 partially abolished proliferation, apoptosis, migration, and invasion induced by miR-26a-5p in CC cells.

**Conclusions:**

MiR-26a-5p alleviated progression of CC by suppressing proliferation, migration and invasion, promoting apoptosis through downregulating HSDL2.

**Supplementary Information:**

The online version contains supplementary material available at 10.1186/s12885-022-09970-x.

## Background

Cervical cancer (CC) was a malignant epithelial tumor in the uterine cervix. It is one of the most common causes of death among women worldwide. The incidence of CC had decreased due to screening early through cytology and virology, and recognizing the important role of human papilloma virus [1, 2]. Until to 2020, it was estimated that approximately 604,127 new cases of CC were diagnosed and 341,831 patients were lethal per year, which was ranked fourth (6.5% for incidence; 7.7% for mortality) in the global incidence of cancer and mortality rate among female [3]. Early CC could be cured by surgery [4]. And the chemotherapy was a mainly strategy for patients with advanced or recurrent CC [5]. The therapeutic effect had been greatly improved because of chemotherapy combining with immunotherapy or Traditional Chinese Medicine therapy, however, the prognosis of patients with CC was unsatisfactory [6–8]. Therefore, it is urgent to find effective prognostic biomarkers and intervention targets, which are of great significance for improving the long-term survival of patients with CC.

MicroRNAs (miRNAs) were 20–24 nucleotides non-coding endogenous RNAs, which downregulated the genes expression by directly targeting to 3’-untranslated regions (3’UTR) of mRNAs and disrupted the stability of mRNAs [9]. Among them, miR-26a-5p had been proved to regulate the development of tumors by reducing the expression of targeted genes. MiR-26a-5p promoted tumor progression by suppressing activation of PTEN signaling in the non-small cell lung cancer [10]. The expression of miR-26a-5p was significantly reduced in endometrial cancer and cell lines, and overexpression of miR-26a-5p enhanced the inhibitory effect of cetuximab by regulating cMet/HGF pathway in vivo and in vitro [11]. The miR-26a-5p/ARPP19 axis modulated nasopharyngeal carcinoma progression by sponging lncRNA SNHG6 [12]. MiR-26a-5p level was low in the gastric cancer and overexpressed miR-26a-5p could promote cell apoptosis, suppress cell proliferation and invasion by inhibiting Wnt5a expression in gastric cancer cells [13]. In conclusion, miR-26a-5p can target a variety of genes to regulate cancer progression and the malignant properties of tumor cells. However, the correlation between miR-26a-5p and tumor progression has not been studied in the CC.

Hydroxysteroid dehydrogenases like 2 (HSDL2) locates in 9q32, includes 12 exons and ubiquitously expresses in fat, liver and other tissues. Numerous studies had shown that the HSDL2 was a key factor of fatty acid regulatory in lipid metabolism [14] and abnormal HSDL2 expression was associated with a variety of cancers, such as bladder cancer[15], breast cancer [16], lung adenocarcinoma [17], thyroid carcinoma [18], cholangiocarcinoma [19], ovarian cancer [20], gliomas [21], pancreatic cancer [14] et al. A large number of studies had shown that abnormal lipid metabolism could accelerate/decelerate the progression of CC [22]. HSDL2 regulated lipid metabolism, and abnormal expression of HSDL2 could promote the malignant characteristics of tumors [14, 23–25], including CC [23]. Meanwhile, it was confirmed that miR-26a-5p was closely related to the progression of CC [26], but its specific molecular regulation mechanism is unclear. Evidence suggested that HSDL2 expression was regulated by miRNAs [27], however, whether miR-26a-5p affect the progression of CC by regulating the expression of HSDL2 needs to further study. In this study, we assessed the relation between the expression of miR-26a-5p and HSDL2, and further investigated the underlying molecular mechanism of miR-26a-5p affecting CC progression. Our findings revealed that miR-26a-5p may be a potential therapeutic target for CC treatment.

## Methods

### Patient samples and ethical approval

The 15 patients were diagnosed CC at department of gynecology in the First people’s Hospital of Huaihua from October 2020 to March 2021. The CC and para-carcinoma (PC) tissues were collected when the patients signed informed consent before surgery. This project was approved by the Medical Research Ethics Committee of Hunan University of Medicine (No. 2020091132) and abided by principles in the Declaration of Helsinki.

### Mirna microarray

The total RNAs were isolated from 6 CC tissues and 6 PC tissues in according to the protocol of TRIzol reagent (15,596,026, Invitrogen, USA) and purified using mirVana™ miRNA Isolation Kit (AM1561, Life Technologies, USA). Then, the differential expression of RNA was identified using miRNA microarray by CapitalBio Corporation (CapitalBio, China).

### Hematoxylin and Eosin (HE) stain

The tissues were fixed using 4% paraformaldehyde for 24 h and embedded in paraffin. The sections of 2–5 μm were cut from the paraffin containing tissues and dewaxed as follow: immersed in xylene twice for 10 min each time, 95% ethanol for 3 min, 85% ethanol for 3 min, 70% ethanol for 3 min. Then, the sections were stained with Hematoxylin for 5–10 min and Eosin for several minutes according to tissues. After dehydrating and permeabilizing, the sections were mounted with neutral resin. Finally, the images were photographed by microscope (Carl Zeiss, GER).

### Antibodies

The antibodies for western blot and immunohistochemistry (IHC) were showed as follow: rabbit anti-HSDL2 antibody (1:200 for IHC; 1:1000 for western blot, 15,631–1-AP, Proteintech, China), β-actin rabbit antibody (1:2000, AF5003, Beyotime, China), goat anti-rabbit IgG (1:1000 for IHC; 1:5000 for western blot, ab6721, abcam, USA).

### IHC analysis

After collecting patient samples, the tissues were embedded into paraffin and cut at 3–5 μm thickness. The heat-induced antigen retrieval was executed at 120 ℃ for 10 min after deparaffinization. The sections were blocked using 5% bovine serum albumin and incubated with anti-HSDL2 antibody at 4 ℃ overnight. Then, the sections were incubated with HRP conjugated anti-rabbit antibody at room temperature (RT) for 0.5–1 h, followed by treatment with 3,3 N-Diaminobenzidine tetrahydrochloride for several seconds. The sections were counterstained with hematoxylin, observed and photographed by a microscope (Olympus, Japan).

### Western blot analysis

The CC tissues, PC tissues and CC cell lines (Hela and C33A) with miRNAs or plasmids were lysed in RIPA buffer (R0020, Solarbio, China) supplemented with 1 mM the protease inhibitor PMSF (P0100, Solarbio, China) on ice for 30 min. Lysates were centrifuged at 12,000 g for 30 min at 4℃. Supernatants were collected and separated by 10% SDS-PAGE. Proteins were transferred onto a polyvinylidene fluoride (PVDF) membrane (Millipore, USA). Based on the molecular weigh of HSDL2 (45 kDa) and β-actin (42 kDa), it was difficult to separate proteins sufficiently to display on the same PVDF membrane. The same samples were transferred on two membranes under exactly the same condition. Meanwhile, the blots of HSDL2 and β-actin were cut prior to hybridization with antibodies at corresponding positions according to the molecular weight of protein Marker. The PVDF membranes were respectively blocked with 5% skilled milk for 1-2 h at RT and incubated with primary antibodies overnight at 4℃, following by incubating with corresponding secondary antibodies at RT for 1–1.5 h. Finally, the protein bands were visualized by SuperSignal ™ West Pico PLUS Chemiluminescent Substrate (#34,579, Thermo, USA). The results were analyzed by ImageJ software (Version 1.8.0, Softnic, USA).

### Cell culture

Normal cervical epithelial cells (GH329) and CC cell lines (C33A, Hela and SiHa) were purchased from American Type Culture Collection and cultured in Dulbecco's Modified Eagle Medium (DMEM, Gibco, USA) supplement with 10% fetal bovine serum (FBS, Gibco, USA) and 1% pencillin-streptomycin (15,140–122, Gibco, USA). The cells were cultured in 5% CO_2_ incubator at 37 ℃.

### HSDL2 plasmids generation and cell transfection

The HSDL2 plasmid was generated using hemo species HSDL2 cDNA ligated into the Xba1 and BamH1 sites of pcDNA3.1. The plasmid with HSDL2 was amplified in *Escherichia coli* and extracted using E.Z.N.A.™ Plasmid Mini Kit (OMEGA, USA) in according to the protocol of manufacture. Constructed plasmids with HSDL2 were validated by sequencing. The miRNA control (miR-NC, 5’-UUCUCCGAACGUGUCACGUTT-3’), miR-26a-5p mimic (mimic, 5’-UUCAAGUAAUCCAGGAUAGGCU-3’) were obtained from GenePharma (Shanghai, China). The plasmid DNAs and miR-NC or mimic were transfected into Hela or C33A cells using Lipofectamine 3000 transfection reagent (L3000015, Themo, USA) in according with manufacture’s protocol.

### Real-time quantitative PCR (RT-qPCR) analysis

Total RNAs of transfected Hela or C33A cells were extracted using TRIzol reagent (R1200-100, Solarbio, China) in according to the protocol of manufacture. Concentration of total RNAs was measured by a NanoDrop 2000 spectrophotometer (Thermo, USA). Then, cDNA was generated by All-One RT MasterMix Kit (G492, abm, Canada) according to manufacturer’s protocol. Finally, the expression miR-26a-5p and HSDL2 were determined using EvaGreen 2 X qPCR MasterMix (MasterMix-S, abm, Canada) according to the manufacturer’s protocol by CFX Connect™ Real-Time System (BIO-RAD, USA) with specific primers which were listed in Table 1. The RT-qPCR was carried out with following parameters: pre-denaturation at 95 ℃ for 10 min; 40 cycles at 95 ℃ for 15 s, 60 ℃ for 1 min. Internal controls were 18S and U6 in RT-qPCR analysis. The expression of target gens was calculated with 2^−ΔΔCt^ method [28].

**Table 1:** Primers of RT-qPCR in this study

Names	Sequences (5’-3’)
MiR-26a-5p Forward primer	TGGGTTCAAGTAATCCAGGA
MiR-26a-5p Reverse primer	TGGTGTCGTGGAGTCG
U6 Forward primer	CTCGCTTCGGCAGCACA
U6 Reverse primer	AACGCTTCACGAATTTGCGT
HSDL2 Forward primer	AAGCCACTCAAGCAATCTATCTG
HSDL2 Reverse primer	GCTCTCCATATCCGACATTCCC
18S Forward primer	AGAAACGGCTACCACATCCA
18S Reverse primer	CACCAGACTTGCCCTCCA

### Proliferation analysis

The proliferation of CC cells was analyzed by CCK-8 assay (C0038, Beyotime, Shanghai, China). Briefly, the transfected cells (5 × 10^3^ cells/well) were seeded into 96-well plate. It was considered 0 h (h) when cells were absolutely adhered. The cells were mixed with CCK-8 solution (10 μl/well) at indicative time, then cultured in a 5% CO2 incubator at 37℃ for 1 h. Finally, the optical density was measured at the wavelength of 450 nm on microplate reader (ReadMax 1900, Flash, Shanghai).

### Apoptosis analysis

The transfected cells (2 × 10^4^ cells/well) were seeded into 24-well plate. The cells were harvested at confluence of 70%, then, stained with Annexin V and PI in according the protocol of Annexin V, FITC Apoptosis Detection Kit (AD10, Dojindo, Japan). Finally, the apoptosis was analyzed using flow cytometry (BD, USA).

### Transwell analysis

The invasion of CC cells was analyzed by transwell analysis. The transwell chambers (MCEP24H48, Millipore, USA) were treated with BD Matrigel (356,234, BD, USA) and medium (Matrigel: medium = 1:8) in incubator for 3 h. Medium was added into 24-well plate. The transwell chambers were arranged in the 24-well plate. The cells at concentration of 5 × 10^4^/ml were seeded into each chamber. The 24-well plate with transwell chamber was placed in the 5% CO_2_ incubator at 37 ℃ for 48 h. The transwell chamber was fixed by 5% paraformaldehyde and stained with 0.1% crystal violet (BL802A, Biosharp, China). The cells in the upper of chamber were scrubbed with swab, and the cells in bottom of chamber were randomly selected to image and counted.

### Wound healing analysis

The migration of CC cells was assessed by wound healing analysis. After transfected 24 h, the cells were seeded in a culture-insert 2 well (180,614/1, ibidi, GER). Cells filling the inner chamber were defined as 0 h. Chamber was removed and the area of images was photographed at regular intervals to analyze migration rate. Migration rate % = (scratch area at 0 h – scratch area at 48 h)/ scratch area at 0 h × 100%.

### Statistical analysis

All data were presented as mean ± SD and analyzed with SPSS23.0 and GraphPad Prism 8.0 software. The *t* test was performed to analyze the difference between two groups. The Kaplan–Meier analysis was operated to analyze OS of patients with CC. The Spearman correlation analysis was used to analyze the correlation between miR-26a-5p and HSDL2 in the patients with CC. All data were obtained from three independent experiments. The *p* value less than 0.05 was considered as statistically significant.

## Results

### Down-regulation of miR-26a-5p indicated a lower overall survival in the patients with CC

**Fig. 1:**
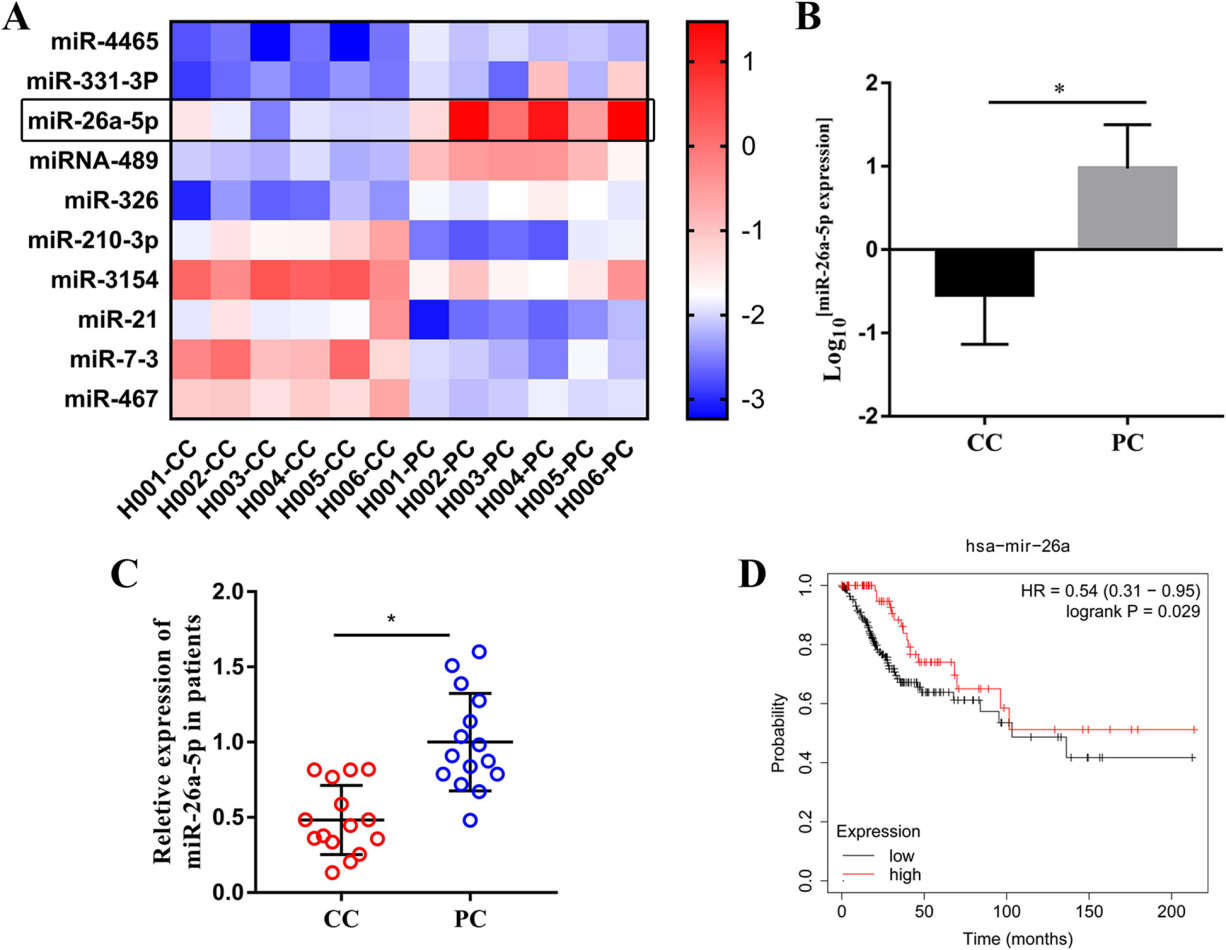
The expression of miR-26a-5p was down-regulated and closely related to OS in the patients with CC. (**A**) The heat map showed the 10 top miRNAs with differential expression in 6 patients with CC by miRNA microarray. The black box represents expression of miR-26a-5p. (**B**) The miR-26a-5p expression of miRNA microarray was statistically analyzed, which was down-regulated in the CC tissues. (**C**) The expression of miR-26a-5p was detected by RT-qPCR analysis in 15 patients with CC, which was showed that the expression of miR-26a-5p in CC tissues was lower than that in PC tissues. (**D**) The OS of patients with CC was obtained online (https://kmplot.com), which indicated down-regulation of miR-26a-5p a lower OS in the patients with CC. All data were from three independence experiments (*p < 0.05, *t*-test). CC: cervical carcinoma; PC: para-carcinoma

Total RNAs of the 6 patients with CC, including CC tissues and PC tissues, were analyzed by miRNA microarray. The 10 top miRNAs with differential expression were showed as Fig. 1A. Among them, the miR-26a-5p expression was significantly downregulated in the CC tissues (Fig. 1A & B). To further explore the miR-26a-5p expression in the patients with CC, the expression of miR-26a-5p was measured in the 15 CC tissues and 15 PC tissues by RT-qPCR. As shown in Fig. 1C, the miR-26a-5p expression of CC tissues was lower than that of PC tissues, which was consistent with the result of miRNA microarray. Then, the OS of patients with CC was analyzed by Kaplan–Meier plotter (https://kmplot.com). The OS of CC patients with low miR-26a-5p expression was shorter than that with high miR-26a-5p expression (Fig. 1D). These results showed that down-regulation of miR-26a-5p indicated an inferior OS in the patients with CC.

### MiR-26a-5p suppressed proliferation and activated apoptosis in CC cells

**Fig. 2:**
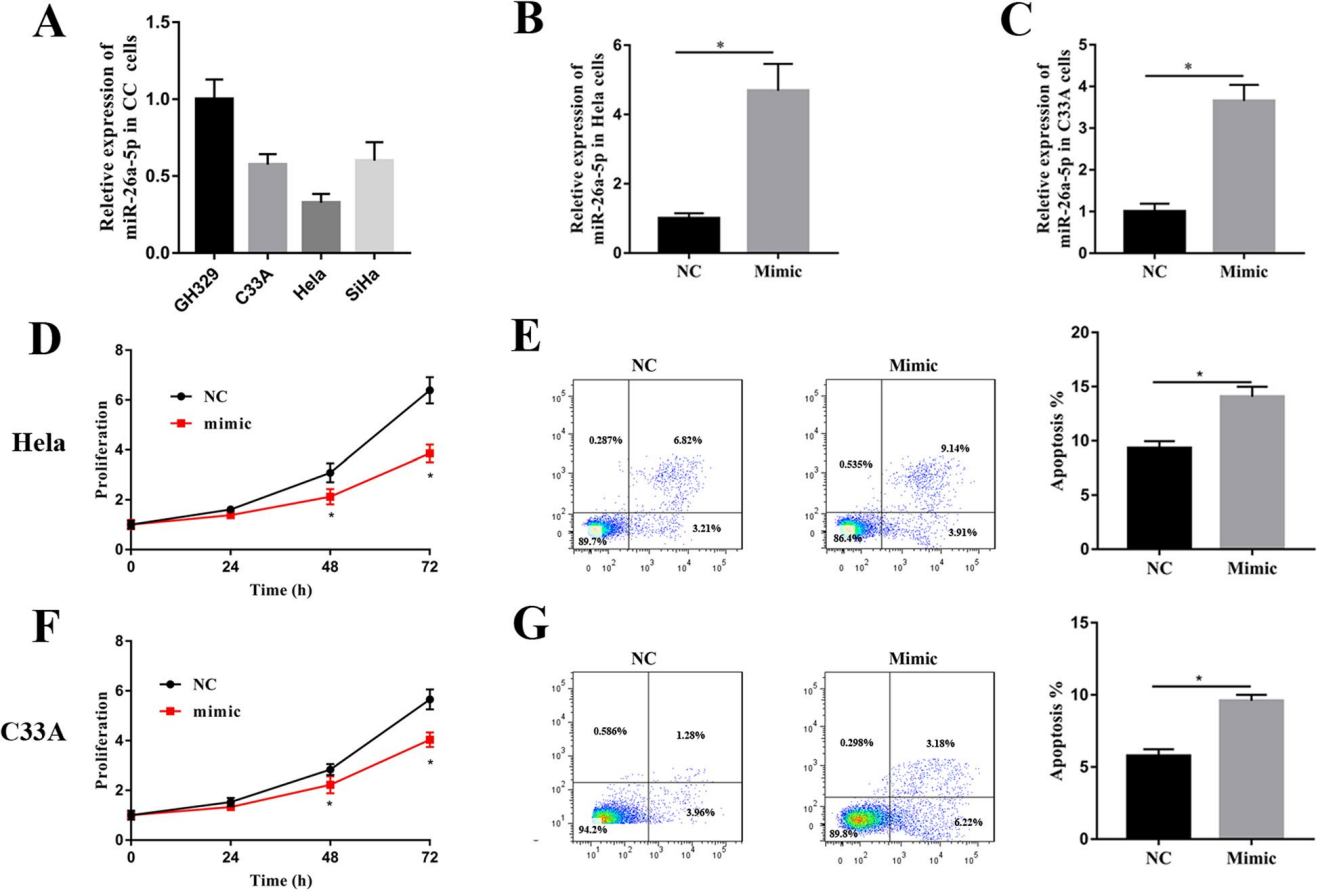
The miR-26a-5p inhibited proliferation and promoted apoptosis in Hela and C33A cells. (**A**) The expression of miR-26a-5p was detected by RT-qPCR in the normal cervical epithelial cells (GH329) and CC cell lines (C33A, Hela and SiHa). The Hela and C33A cells were transfected with miR-26a-5p mimic or miR-NC. The expression of miR-26a-5p was measured in the transfected Hela (**B**) and C33A cells (**C**). The proliferation of Hela (**D**) and C33A (**F**) cells transfected with miR-NC or miR-26a-5p mimic was analyzed by CCK-8 analysis. The apoptosis of Hela (**E**) and C33A (**G**) cells transfected with miR-NC or miR-26a-5p mimic was analyzed by flow cytometry. All data were from three independence experiments (*p < 0.05, *t*-test). NC: miRNA control; Mimic: miR-26a-5p mimic

To investigate the effect of miR-26a-5p on the malignant properties of CC cells, the expression of miR-26a-5p was detected by RT-qPCR in GH329, C33A, Hela and SiHa cells. Compared with GH329, the expression of miR-26a-5p was down-regulated in the CC cell lines (Fig. 2A). According to expression of miR-26a-5p, the C33A and Hela cells were selected to explore the effect of miR-26a-5p on the malignant properties, including proliferation, apoptosis, migration and invasion. The Hela and C33A cells were transfected miR-NC or miR-26a-5p mimic. Then, the miR-26a-5p expression of Hela and C33A cells with miR-26a-5p mimic was obviously higher than those with miR-NC, which indicated that miR-26a-5p was successfully expressed in HeLa and C33A cells (Fig. 2B & C). The proliferation was assessed by CCK-8 analysis, the proliferation of Hela cells with miR-26a-5p mimic was significantly inhibited after transfection for 48 h and 72 h (Fig. 2D). The apoptosis was measured by flow cytometry. As shown in Fig. 2E, the apoptosis of Hela with miR-26a-5p mimic was dramatically increased compared with that of Hela with miR-NC (Fig. 2E). The results of C33A cells with miR-26a-5p mimic were consistent with those of Hela with miR-26a-5p mimic, which showed that miR-26a-5p inhibited proliferation, promoted apoptosis in the C33A cells (Fig. 2F & G).

### MiR-26a-5p reduced migration and invasion in the CC cells

**Fig. 3:**
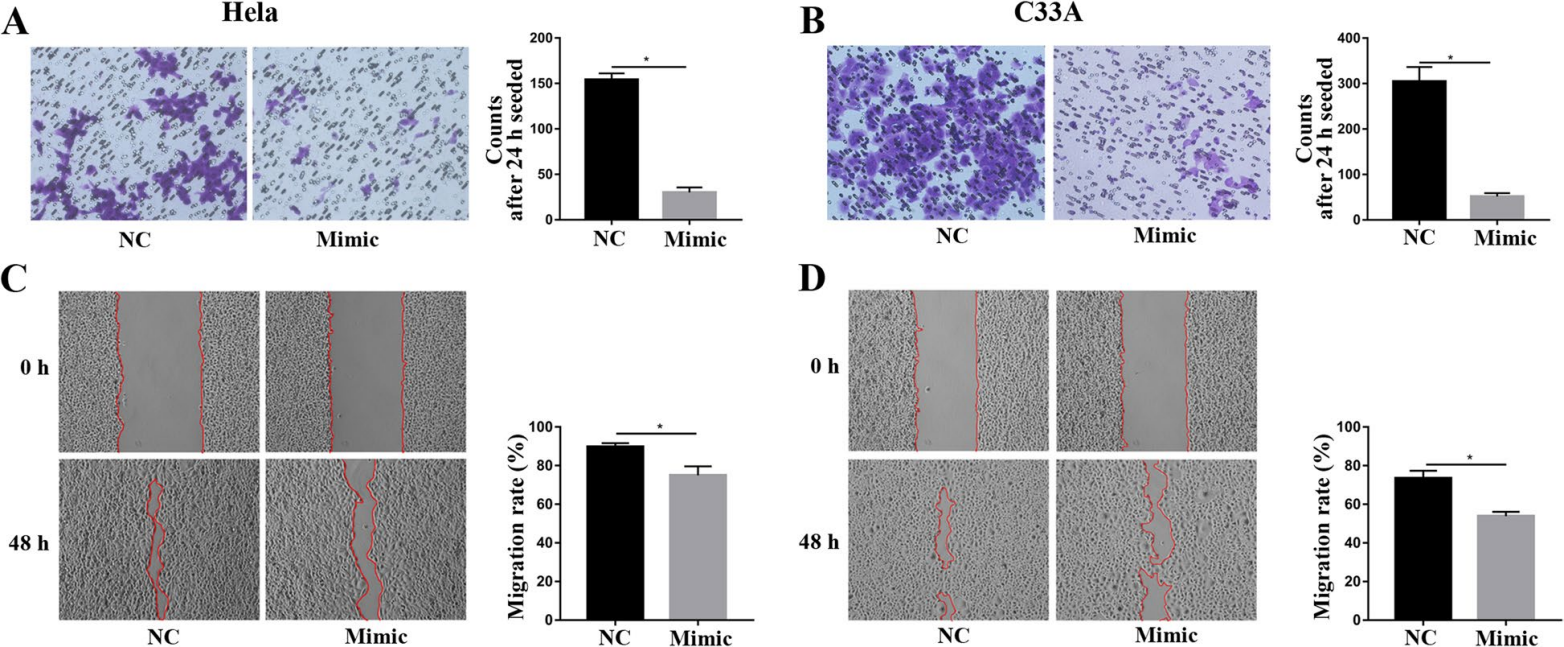
The miR-26a-5p inhibited invasion and migration in the Hela and C33A cells. The Hela and C33A cells were transfected miR-NC or miR-26a-5p mimic. The invasion was analyzed by transwell analysis in the Hela (**A**) and C33A (**B**) cells. The migration was assessed by wound healing in the Hela (**C**) and C33A (**D**) cells. All data were from three independence experiments (*p < 0.05, *t*-test)

After seeding 24 h, the number of cells that penetrated the transwell chamber was significantly decreased in the Hela and C33A cells transfected miR-26a-5p mimic compared with Hela transfected miR-NC, which suggested miR-26a-5p inhibited invasion of Hela and C33A cells (Fig. 3A and B).

The Hela and C33A cells were transfected with miR-NC or miR-26a-5p mimic. After scratch for 48 h, the area of the wound was increased in the CC cells transfected miR-26a-5p mimic, compared with that in the CC cells transfected miR-NC, which indicated that the migration rate of the Hela and C33A cells transfected miR-26a-5p mimic were suppressed (Fig. 3C and D).

### MiR-26a-5p directly regulated the expression of HSDL2 in CC cells

**Fig. 4:**
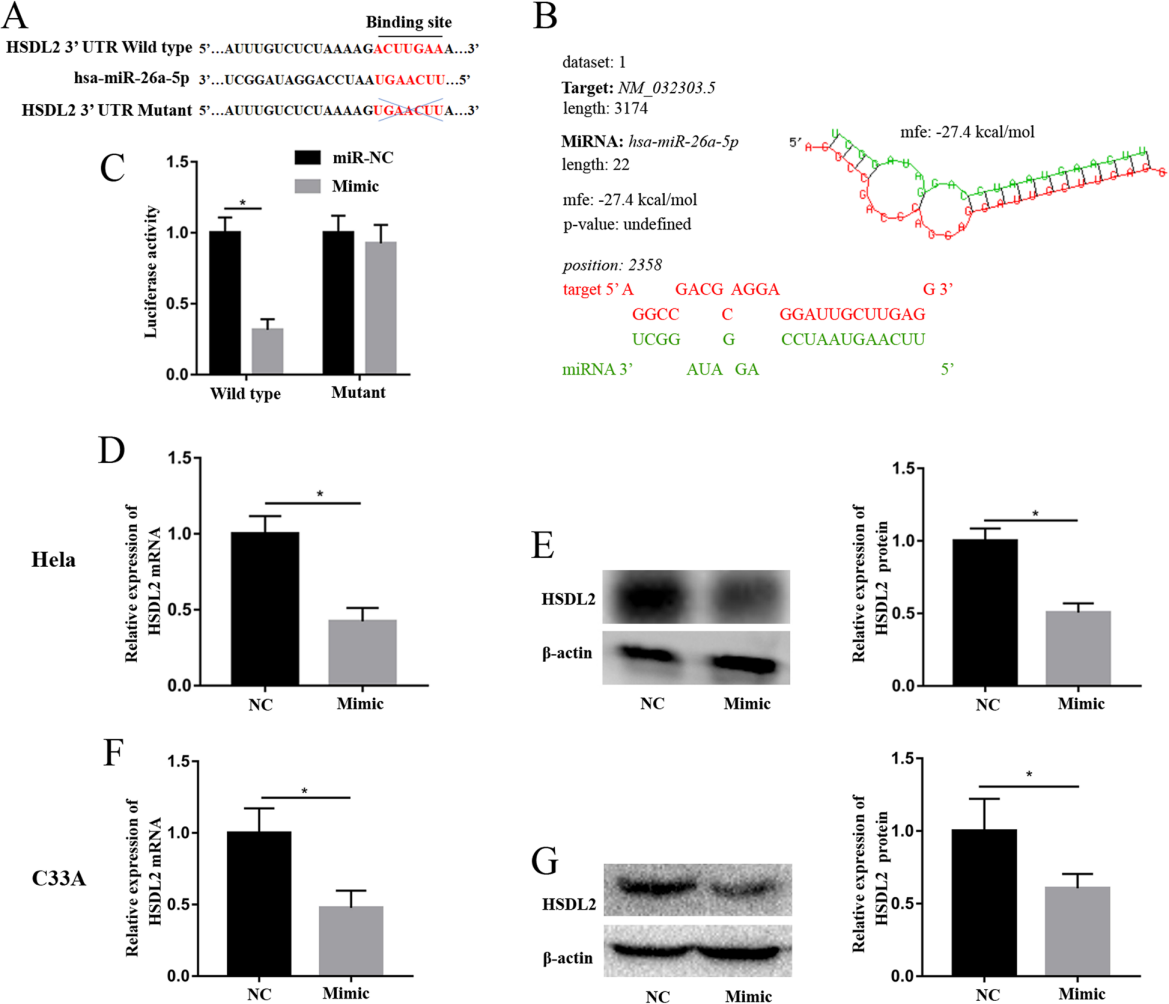
The miR-26a-5p directly regulated the expression of HSDL2 in the Hela and C33A cells. (**A**) Sequence of HSDL2 3’-UTR binding to the miR-26a-5p was predicted by TargetScan and miRDB database. (**B**) The free energy score for binding between miR-26a-5p and HSDL2 mRNA was calculated using RNAhybird database. (**C**) Luciferase activity was analyzed in the Hela cells transfected miR-NC or miR-26a-5p mimic and HSDL2 3’-UTR wild type or mutant. The expression of HSDL2 mRNA and HSDL2 protein were analyzed in the Hela (**D** and **E**) and C33A cells (**F** and **G**) transfected miR-NC or miR-26a-5p mimic. All data were from three independence experiments (*p < 0.05, *t*-test)

Bioinformatics and luciferase reporter assay were performed to investigate whether miR-26a-5p directly regulated HSDL2 expression. Analysis of data in TargetScan (http://www.targetscan.org/) and miRDB (http://mirdb.org/) revealed that miR-26a-5p directly binds the HSDL2 3’-UTR (Fig. 4A). Based on RNAhybird database (http://bibiserv.techfak.uni-bielefeld.de/rnahybrid), the free energy score for binding between miR-26a-5p and HSDL2 mRNA was -27.4 kcal/mol (Fig. 4B), confirming the direct binding between the two molecules. Luciferase activity assay revealed that miR-26a-5p mimic substantially modulated luciferase activity of Hela cells transfected with HSDL2 3’-UTR wild type, whereas the luciferase activity of the cells over-expressing miR-26a-5p and HSDL2 3’-UTR mutant was comparable to that of cells over-expressing miR-NC and HSDL2 3’-UTR mutant (Fig. 4C). Further RT-qPCR and western blot analyses revealed that over-expression of miR-26a-5p significantly decreased expression of HSDL2 mRNA and protein, relative to miR-NC in the Hela (Fig. 4D and E) and C33A cells (Fig. 4F and G).

These findings demonstrated that miR-26a-5p directly regulated the expression of HSDL2 in the CC cells.

### The expression of HSDL2 was negatively correlated with miR-26a-5p in patients with CC

**Fig. 5:**
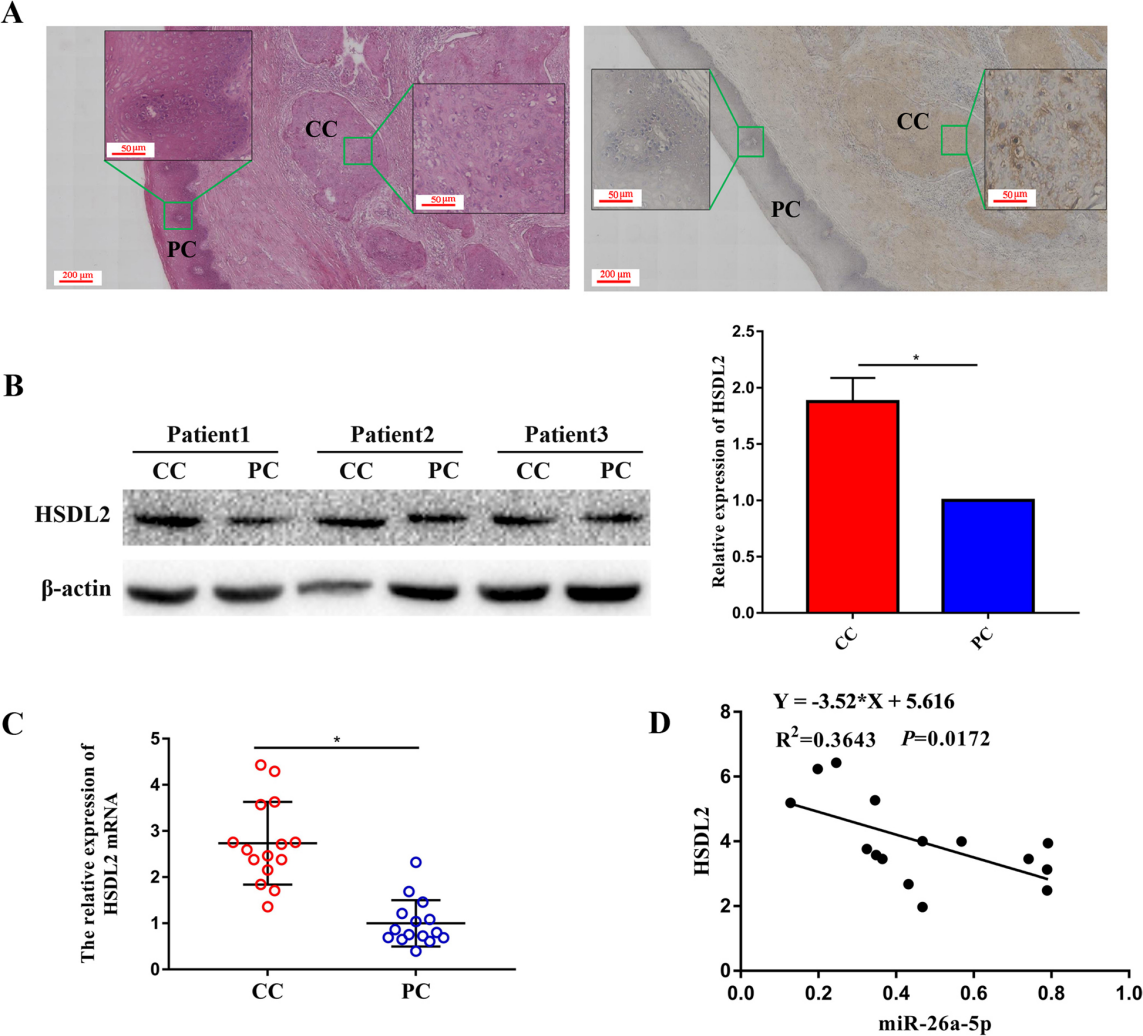
HSDL2 was upregulated and negatively correlated with miR-26a-5p in patients with CC. (**A**) The CC tissues and PC tissues was identified by HE stains (Left) and the expression of HSDL2 was analyzed by IHC (Right). (**B**) The expression of HSDL2 protein was measured by western blot analysis in the 3 patients with CC. (**C**) The expression of HSDL2 mRNA was detected by RT-qPCR in the 15 patients with CC. (**D**) The correlation between the expression of HSDL2 and miR-26a-5p was analyzed by Spearman correlation analysis. All data were from three independence experiments (*p < 0.05, *t*-test)

In order to explore the expression of HSDL2 in CC tissues and PC tissues, HE and IHC were performed. As shown in Fig. 5A, the yellow–brown particles were abundant in the cells of CC tissue, however, absent in the cells of PC tissue, which suggested that expression of HSDL2 was obviously increased in CC tissues. Three patients with CC were randomly selected to measure the expression of HSDL2 protein by western blot analysis. The result showed that the expression of HSDL2 protein was increased in the CC tissues (Fig. 5B). The expression of HSDL2 mRNA was verified in the 15 patients with CC by RT-qPCR. Consistent with IHC and western blot results, the expression of HSDL2 was up-regulated in CC (Fig. 5C). Then, correlation analysis results showed that the expression of HSDL2 was increased in the CC patients with low miR-26a-5p expression, and decreased of HSDL2 was displayed in the patients with high expression of miR-26a-5p, which suggested that the expression of HSDL2 was negatively correlated with miR-26a-5p in the patients with CC (Fig. 5D).

### MiR-26a-5p inhibited proliferation, invasion and migration, promoted apoptosis by downregulation of HSDL2 in CC cells

**Fig. 6:**
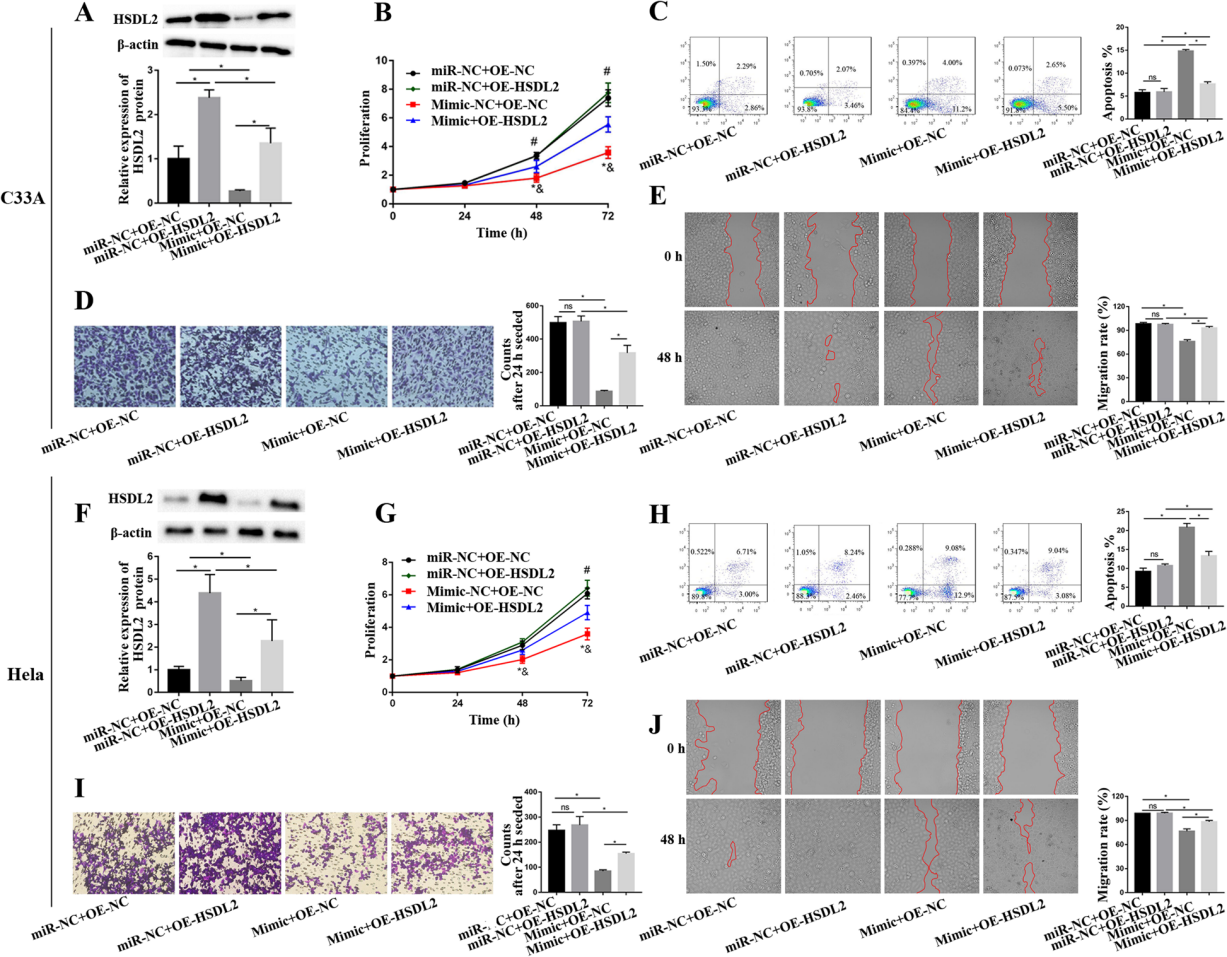
HSDL2 weakened the reduction of proliferation, invasion and migration, enhancement of apoptosis by miR-26a-5p in the Hela and C33A cells. (**A-E**) The HSDL2 expression, proliferation, apoptosis, invasion and migration were respectively detected by WB, CCK-8, flow cytometry, transwell and wound healing analysis in the C33A cells transfected miR-NC or miR-26a-5p and OE-NC or OE-HSDL2. (**F-J**) The HSDL2 expression, proliferation, apoptosis, invasion and migration were respectively detected by WB, CCK-8, flow cytometry, transwell and wound healing analysis in the Hela cells transfected miR-NC or miR-26a-5p and OE-NC or OE-HSDL2. All data were from three independence experiments (* vs miR-NC + OE-NC, *p* < 0.05; & vs Mimic + OE-HSDL2, *p* < 0.05; # vs Mimic + OE-HSDL2, *p* < 0.05, *t*-test for proliferation analysis; ns *p* > 0.05, *p < 0.05, *t*-test for HSDL2 expression, apoptosis, invasion and migration analyses)

The rescue experiments were performed to verify miR-26a-5p regulating proliferation, apoptosis, invasion and migration by inhibiting the expression of HSDL2 in the CC cells. Hela and C33A cells were co-transfected miR-NC or miR-26a-5p mimic and control plasmids (pcDNA3.1, OE-NC) or HSDL2 plasmids (OE-HSDL2). The HSDL2 expression of C33A cells was showed as Fig. 6A, compared with C33A cells with miR-NC + OE-NC, the expression of HSDL2 was significantly upregulated in cells with miR-NC + OE-HSDL2, and the expression of HSDL2 was dramatically downregulated in cell with miR-26a-5p mimic + OE-NC. The HSDL2 expression of cells with miR-26a-5p mimic + OE-HSDL2 was decreased, relative to that of cells with miR-NC + OE-HSDL2. Compared with C33A cells co-transfected miR-NC + OE-NC, the proliferation of C33A cells co-transfected miR-26a-5p mimic + OE-NC was significantly reduced, whereas the proliferation of cells co-transfected miR-26a-5p mimic + OE-HSDL2 was increased, relative to that of cells co-transfected miR-26a-5p mimic + OE-NC. No significant difference in cell proliferation between cells with miR-NC + OE-NC and cells with miR-NC + OE-HSDL2; however, the proliferation of cells with miR-26a-5p mimic + OE-HSDL2 was observably inhibited, compared with cells co-transfected miR-NC + OE-HSDL2. Results of proliferation suggested that HSDL2 partly abolished inhibition of proliferation by miR-26a-5p (Fig. 6B). Flow cytometry analysis revealed that apoptosis of C33A cells transfected miR-26a-5p mimic + OE-NC was dramatically higher than that of cells transfected miR-NC + OE-NC, however, the apoptosis of the cells transfected miR-26a-5p mimic and OE-HSDL2 was lower than that of cells transfected miR-26a-5p mimic and OE-NC. The apoptosis was consistent between miR-NC + OE-HSDL2 cells and miR-NC + OE-NC cells, but, the apoptosis of cells with miR-26a-5p mimic + OE-HDSL2 was increased compared with cells co-transfected miR-NC + OE-HSDL2. These results indicated HSDL2 retarded induction of apoptosis by miR-26a-5p (Fig. 6C). The number of invasion and migration rate were the same between miR-NC + OE-HSDL2 and miR-NC + OE-NC C33A cells, the invasion and migration rate were decreased in the C33A cells transfected miR-26a-5p mimic + OE-NC, relative to those in the cells transfected miR-NC + OE-NC, nevertheless, the invasion and migration rate were increased in the cells transfected miR-26a-5p mimic + OE-HSDL2 compared with those in the cells transfected miR-26a-5p mimic + OE-NC, the invasion and migration rate of C33A cells with miR-26a-5p mimic + OE-HSDL2 were reduced compared with those of cells with miR-NC + OE-HSDL2, which showed that HSDL2 rescued decreasing of invasion and migration by miR-26a-5p (Fig. 6D and E). Meanwhile, the HSDL2 expression, proliferation, apoptosis, invasion and migration were respectively detected in the Hela cells transfected miR-NC or miR-26a-5p mimic and OE-NC or OE-HSDL2, and the results of Hela cells were consistent with those of C33A cells, which showed that HSDL2 alleviated the effect of miR-26a-5p on proliferation, apoptosis, invasion and migration (Fig. 6F-J).

## Discussion

The role of miRNAs had been attracted much attention in tumor and evidences showed that the abnormal expression of miRNAs was closely related to the occurrence and development of CC. The miR-802 was significantly downregulated in CC samples as well as cells, and inhibited the growth and aggressiveness of CC cell by targeting MYLIP [29]. Yang [30] et al.demonstrated that miR-362 could work as an anti-oncomiR that suppressed proliferation and promoted apoptosis in CC cells via BAP31 and TGFβ/Smad pathway. Wei [31] et al. verified that miR-34c-5p targeted Notch 1 and inhibited the metastasis and invasion of CC. It was reported that miR-381 targeted *GPR34* to regulate the growth, migration and invasion of human CC cells [32]. It was seen that the miRNAs regulated malignant properties of the CC cells by downregulating targeted genes. In our study, the downregulation of miR-26a-5p was first identified by miRNA microarray and RT-qPCR, meanwhile indicated a lower overall survival in the patients with CC. Moreover, overexpression of miR-26a-5p distinctly suppressed proliferation, migration and invasion, promoted apoptosis in the Hela and C33A cells.

Previous studies had shown that abnormal expression of HSDL2 was associated with a variety of tumors. The expression of HSDL2 was upregulated in the human bladder cancer cell lines, and HSDL2 knockdown inhibited bladder cancer progression by reducing proliferation, promoting apoptosis in vitro or vivo [15]. The high expression of HSDL2 was presented in breast cancer tissues, related to high histological grades, late clinical stages and lower overall survival, even depletion of HSDL2 inhibited proliferation and induced cell cycle arrest in breast cancer [16]. Shi’s researches suggested that HSDL2 was upregulated in the lung adenocarcinoma tissue and HSDL2 knockdown inhibited lung adenocarcinoma progression via downregulating AKT2 expression [17]. HSDL2 was in highly expressed in human ovarian cancer and was positively correlated with tumor progression and lymphatic metastasis, meanwhile, HSDL2 knockdown inhibited tumorigenesis in vivo or vitro [20]. HSDL2 was highly expressed in pancreatic cancer and connected with shorter overall survival, meanwhile proliferation and lipid metabolism were further inhibited when HSDL2 was silenced in pancreatic cancer cell [14]. In this study, we detected the expression of HSDL2 in patients with CC. Our results were consistent with a previous research, which HSDL2 was high expressed in the patients with CC [23]. Then, the correlation between the expression of HSDL2 and miR-26a-5p was analyzed in the CC patients. The correlation analysis results showed that the expression of HSDL2 was negatively correlated with the expression of miR-26a-5p.

The further study was performed to investigate underly mechanism of miR-26a-5p on inhibiting proliferation, invasion and migration, and promoting apoptosis in the Hela and C33A cells. Bioinformatics and luciferase reporter assay verified that miR-26a-5p directly targeted HSDL2. The expression of HSDL2 was downregulated in the CC cells with transfected miR-26a-5p mimic. Unexpectedly, proliferation, invasion and migration were inappreciably reduced; apoptosis was slightly increased in the Hela and C33A cells with miR-NC + OE-HSDL2, relative to cell with miR-NC + OE-NC, which seemed inconsistent with the upregulation of HSDL2 in CC patients. We speculate that the reason is as follow: CC cells, such Hela and C33A originally express higher HSDL2, which lead to malignant characteristics of CC cells. The expression of HSDL2 is insignificant increased in the CC cells overexpressed HSDL2 due to originally higher expression of HSDL2. So, the promotion of malignant characteristics is difficultly detected in a shorter period of time. Nevertheless, the rescue experiments showed overexpression of HSDL2 partly reversed the inhibition of proliferation, invasion and migration, and stimulation of apoptosis induced by miR-26a-5p, which demonstrated that the miR-26a-5p retarded proliferation, invasion and migration, and accelerated apoptosis via regulating HSDL2 in the CC cells. Previous studies had shown that miR-26a-5p targeted different genes to regulate the malignant potential of multiple tumors resulted in alleviating/promoting progression of cancer [33–36]. Our study firstly proved that miR-26a-5p inhibited proliferation, invasion and migration, promoted apoptosis by downregulating the expression of HSDL2 in the CC cells. Combined with Yang’s research [23], we conclude that miR-26a-5p suppresses proliferation, invasion and migration, promotes apoptosis though inhibiting Epithelial-mesenchymal transition (EMT) by targeting HSDL2 in the CC cells, which supplemented the mechanism of miR-26a-5p-HSDL2-EMT on influencing CC progression.

## Conclusions

In this study, the expression of miR-26a-5p was decreased and down-regulation of miR-26a-5p indicated a poor overall survival in the patients with CC. Overexpression of miR-26a-5p alleviated CC progress through modulating the malignant properties by reducing the expression of HSDL2 in vitro. Accordingly, intervention of miR-26a-5p/HSDL2 is potential therapeutic strategy for CC treatment. Even so, the molecular mechanism of miR-26a-5p/HSDL2 on influencing CC progression is further explored in the further.

## Supplementary Information

Additional file 1. miR-26a-5p regulates proliferation, apoptosis, migration and invasion via inhibiting hydroxysteroid dehydrogenase like-2 in cervical cancer cell.

## Data Availability

All relevant data are included in the manuscript and its associated files.

## Abbreviations

CC: Cervical cancer
CCK-8: Cell Counting Kit-8
DMEM: Dulbecco's Modified Eagle Medium
FBS: Fetal bovine serum
HE: Hematoxylin and Eosin
HSDL2: Hydroxysteroid dehydrogenase like-2
IHC: Immunohistochemistry
MiRNAs: MicroRNAs
NC: Normal control
OE: Overexpression
PC: Para-carcinoma
PVDF: Polyvinylidene fluoride
RT: Room temperature
RT-qPCR: Real-time quantitative PCR
3’UTR: 3’-Untranslated regions
