# Supplementary material for: MiR-26a-5p regulates proliferation, apoptosis, migration and invasion via inhibiting hydroxysteroid dehydrogenase like-2 in cervical cancer cell
Source: BMC Cancer. 2022 Aug 10;22:876. doi: 10.1186/s12885-022-09970-x (PMC9367141; doi:10.1186/s12885-022-09970-x)
Supplement: Supplementary file 1 — Additional file 1. miR-26a-5p regulates proliferation, apoptosis, migration and invasion via inhibiting hydroxysteroid dehydrogenase like-2 in cervical cancer cell. [file 12885_2022_9970_MOESM1_ESM.doc]

**miR-26a-5p regulates proliferation, apoptosis, migration and invasion via inhibiting hydroxysteroid dehydrogenase like-2 in cervical cancer cell**

**The original blots shown in the revised Figure 4E were listed as follow:**


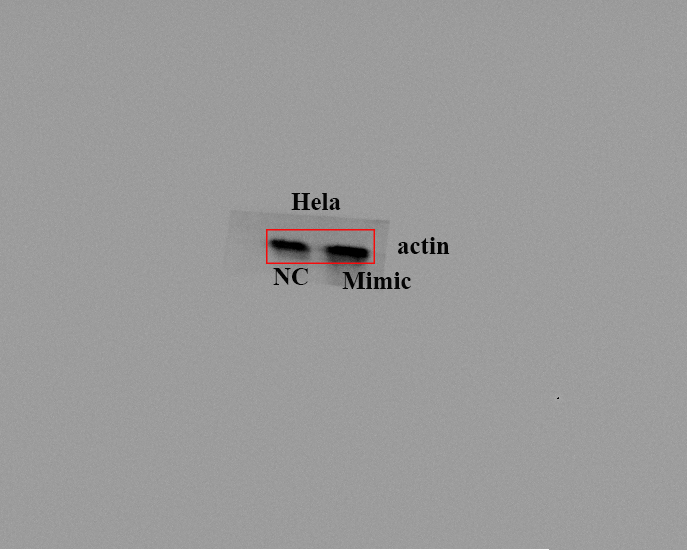

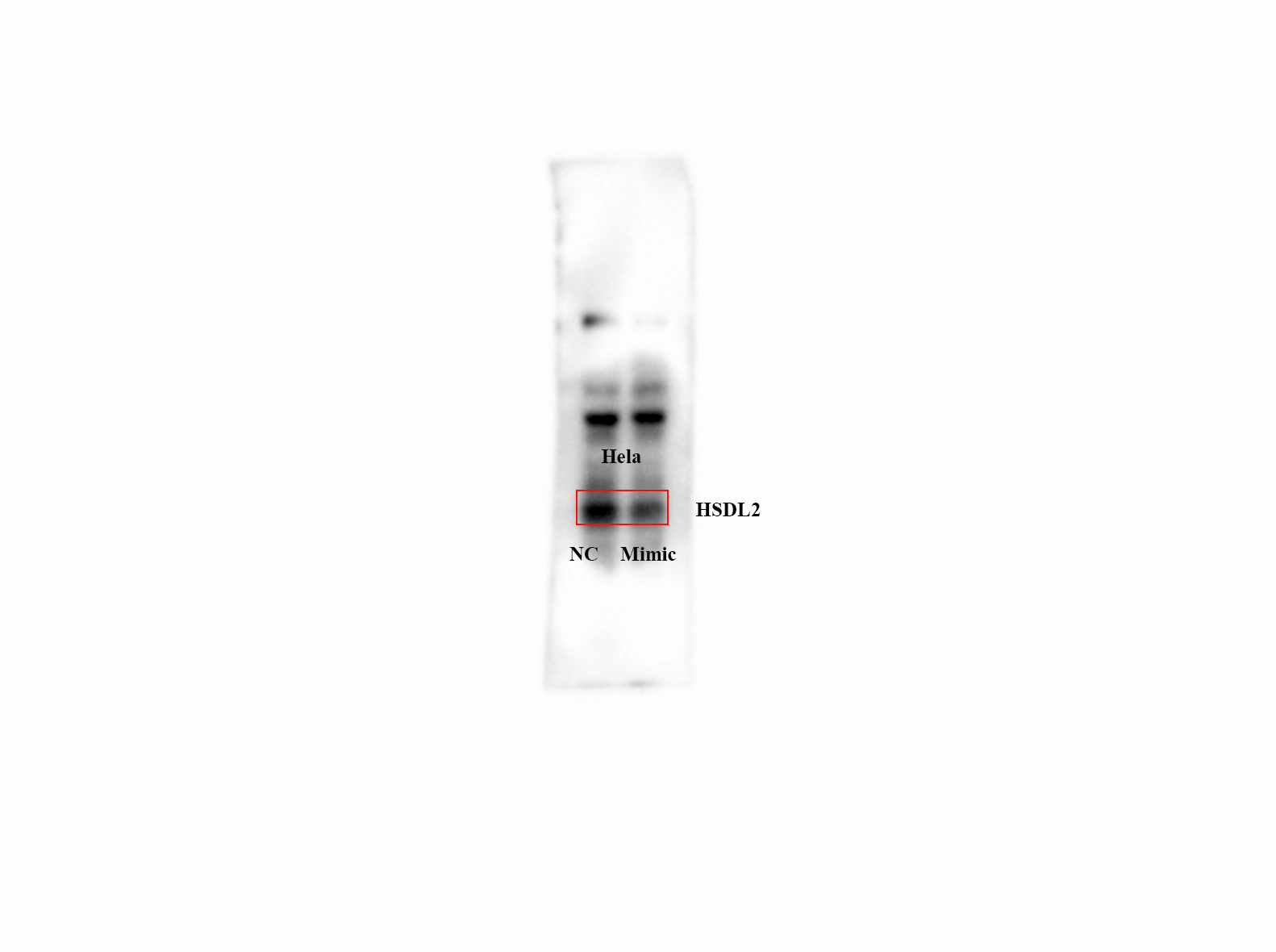


The red boxes show the cropped edge.

**The replicate images of blots in the Hela cells were listed as follow.**

**Replicate 1:**


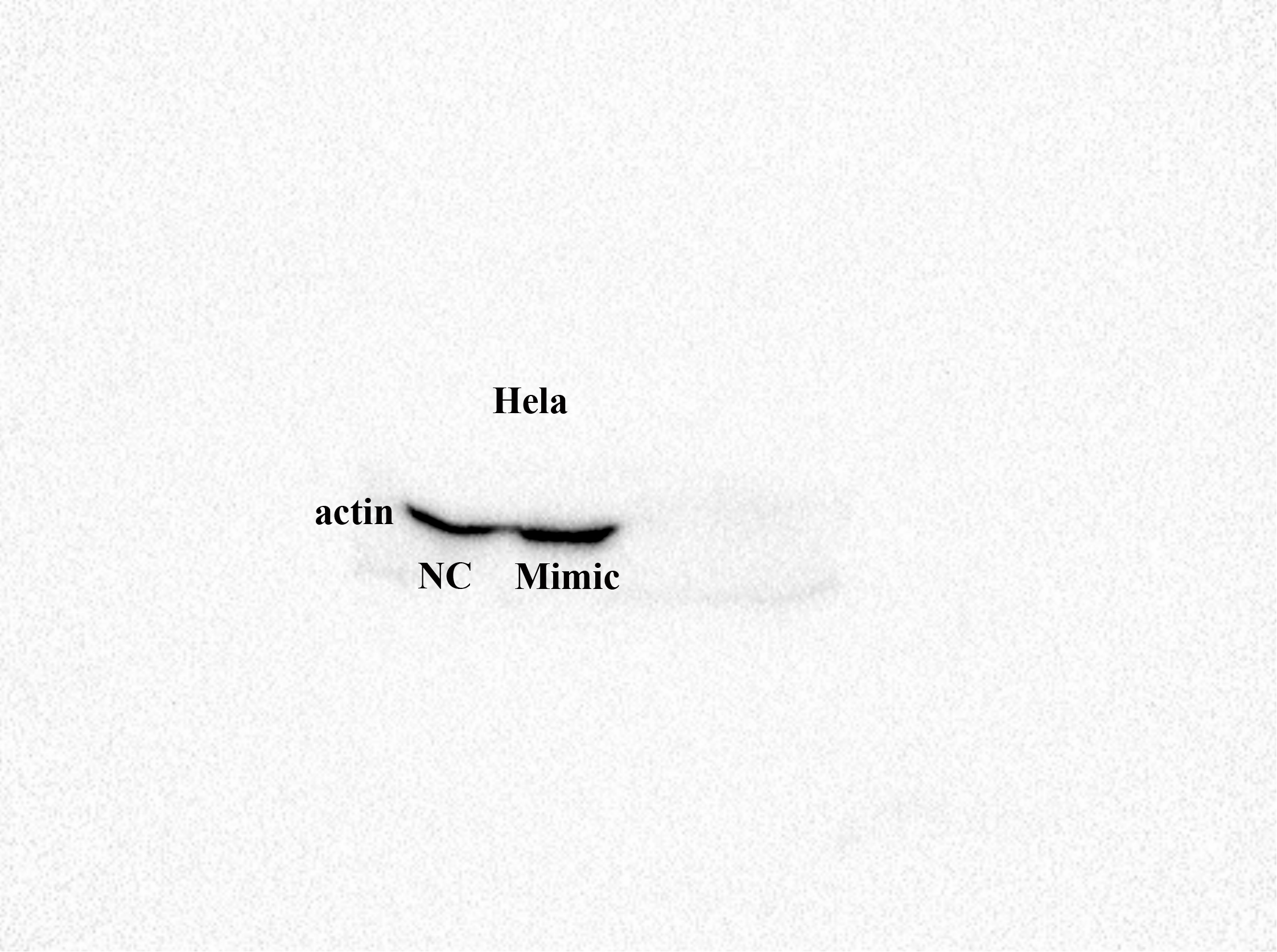

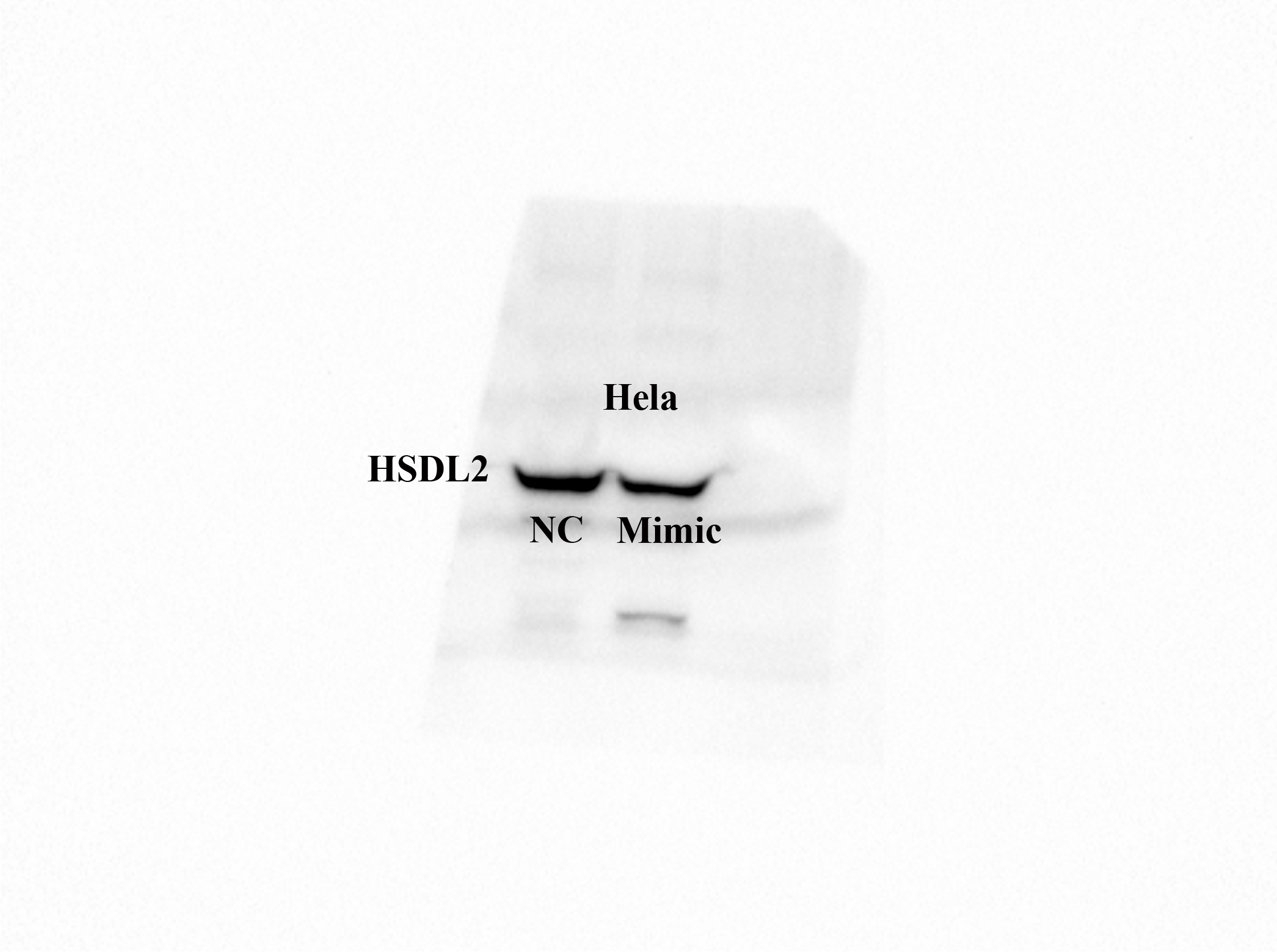


**Replicate 2:**


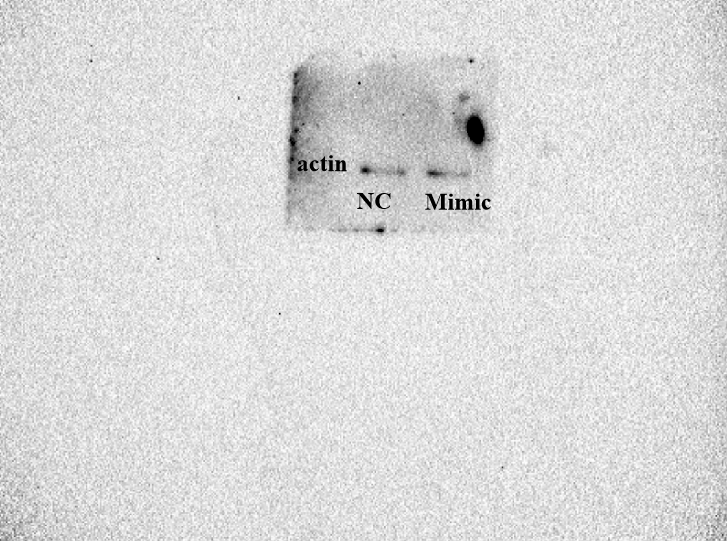

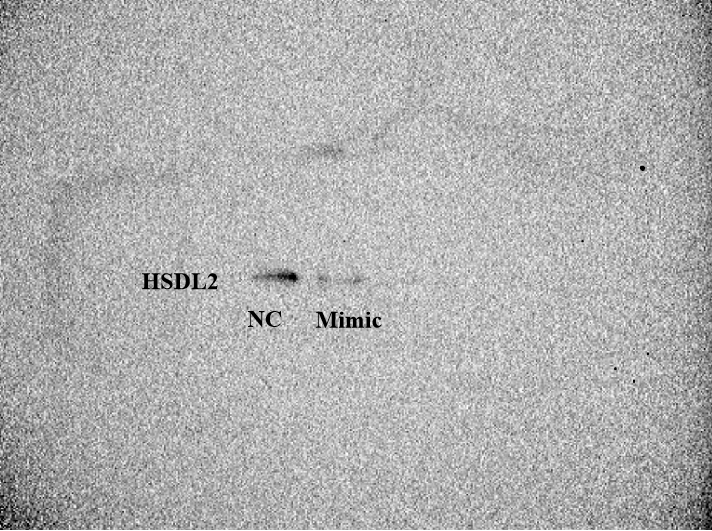


**The original blots shown in the revised Figure 4G were listed as follow:**


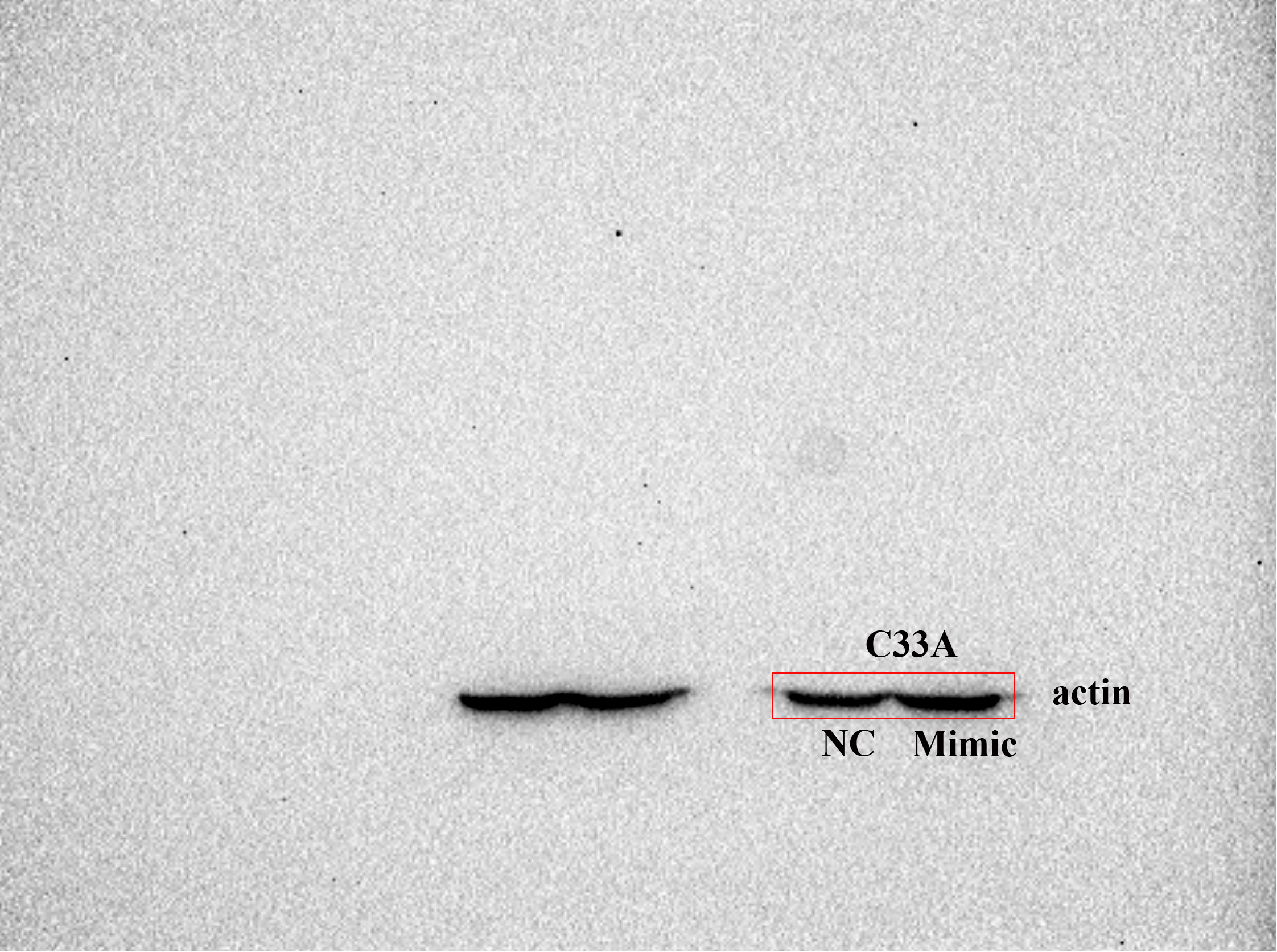

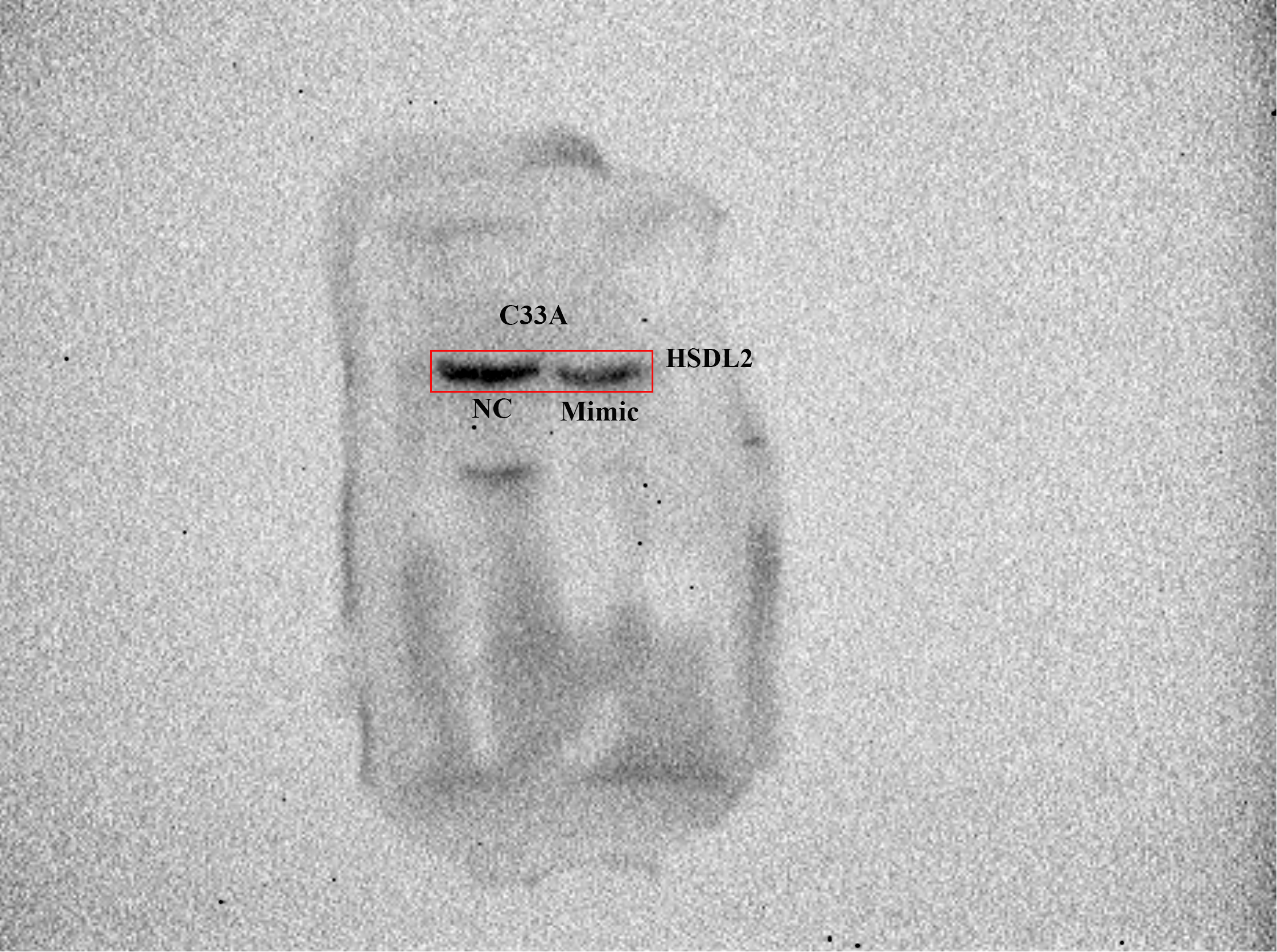


The red boxes show the cropped edge.

**The replicate images of blots in the C33A cells were listed as follow.**

**Replicate 1:**


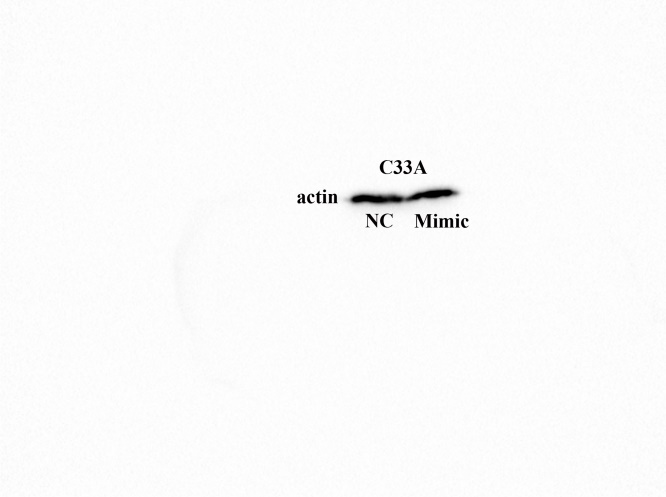

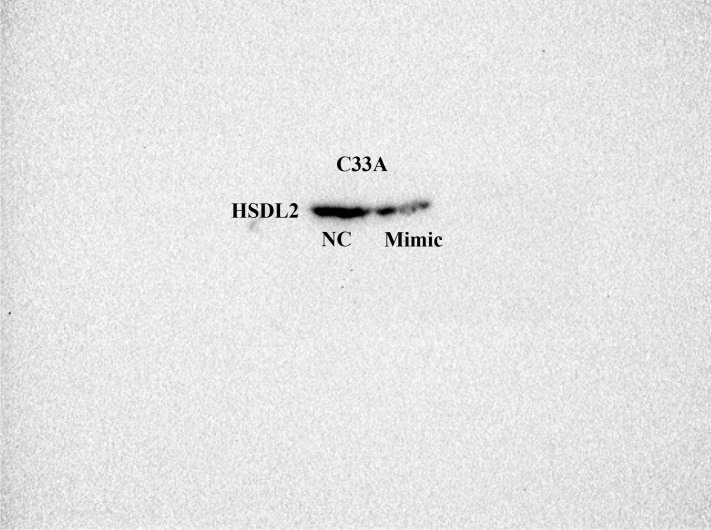


**Replicate 2:**


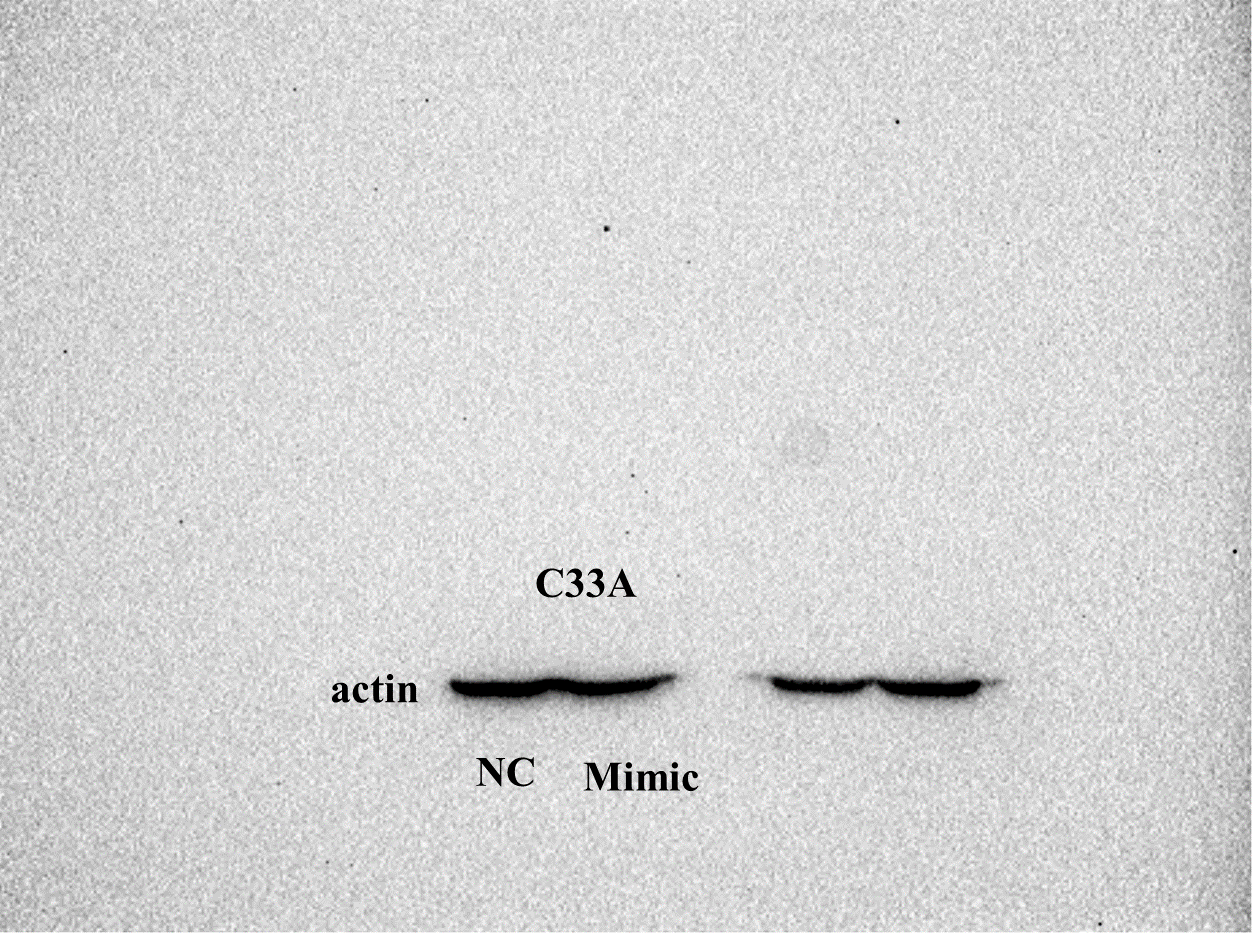

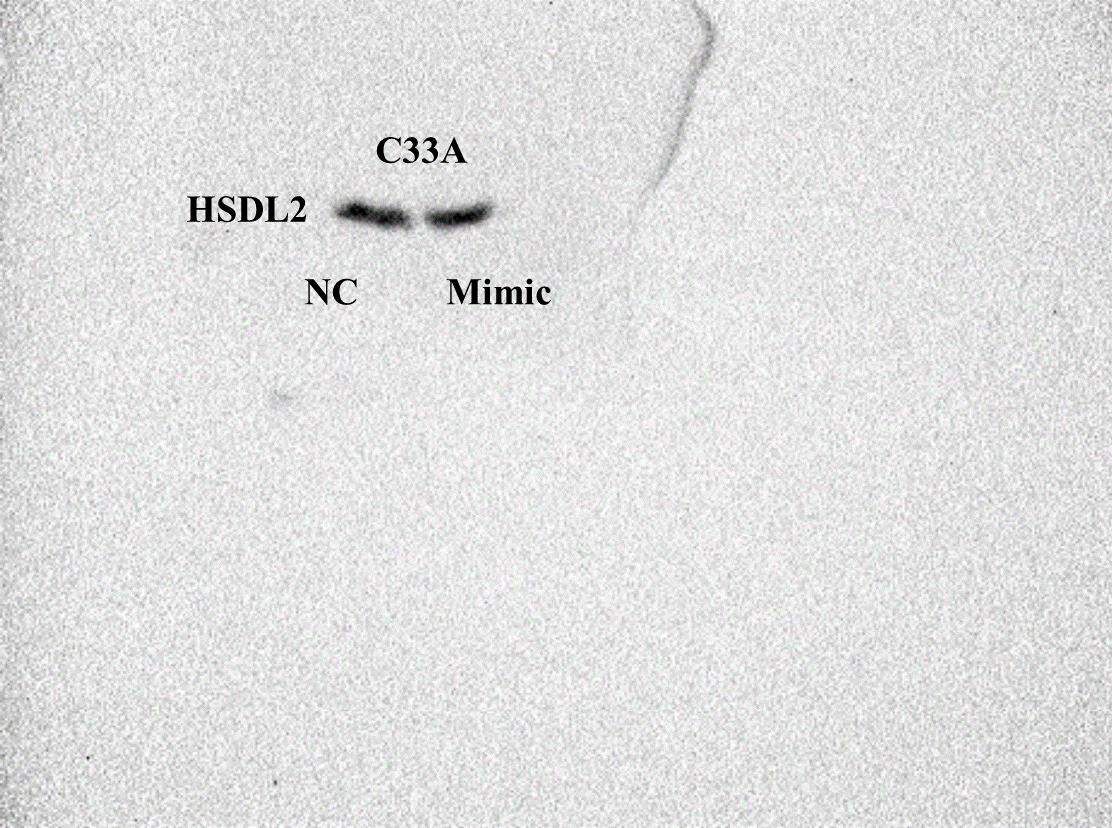


**The original blots shown in the revised Figure 5B were listed as follow:**


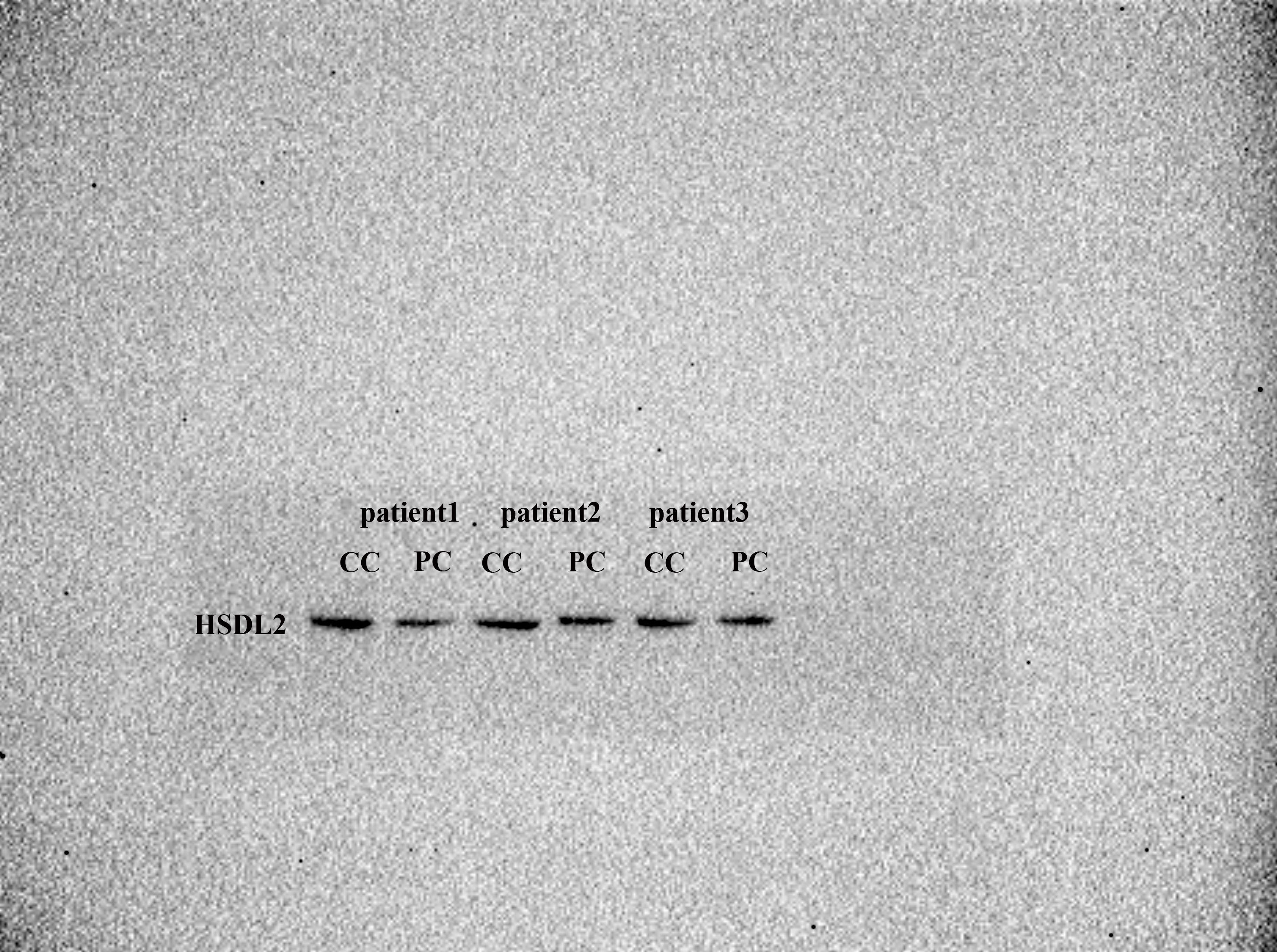
**
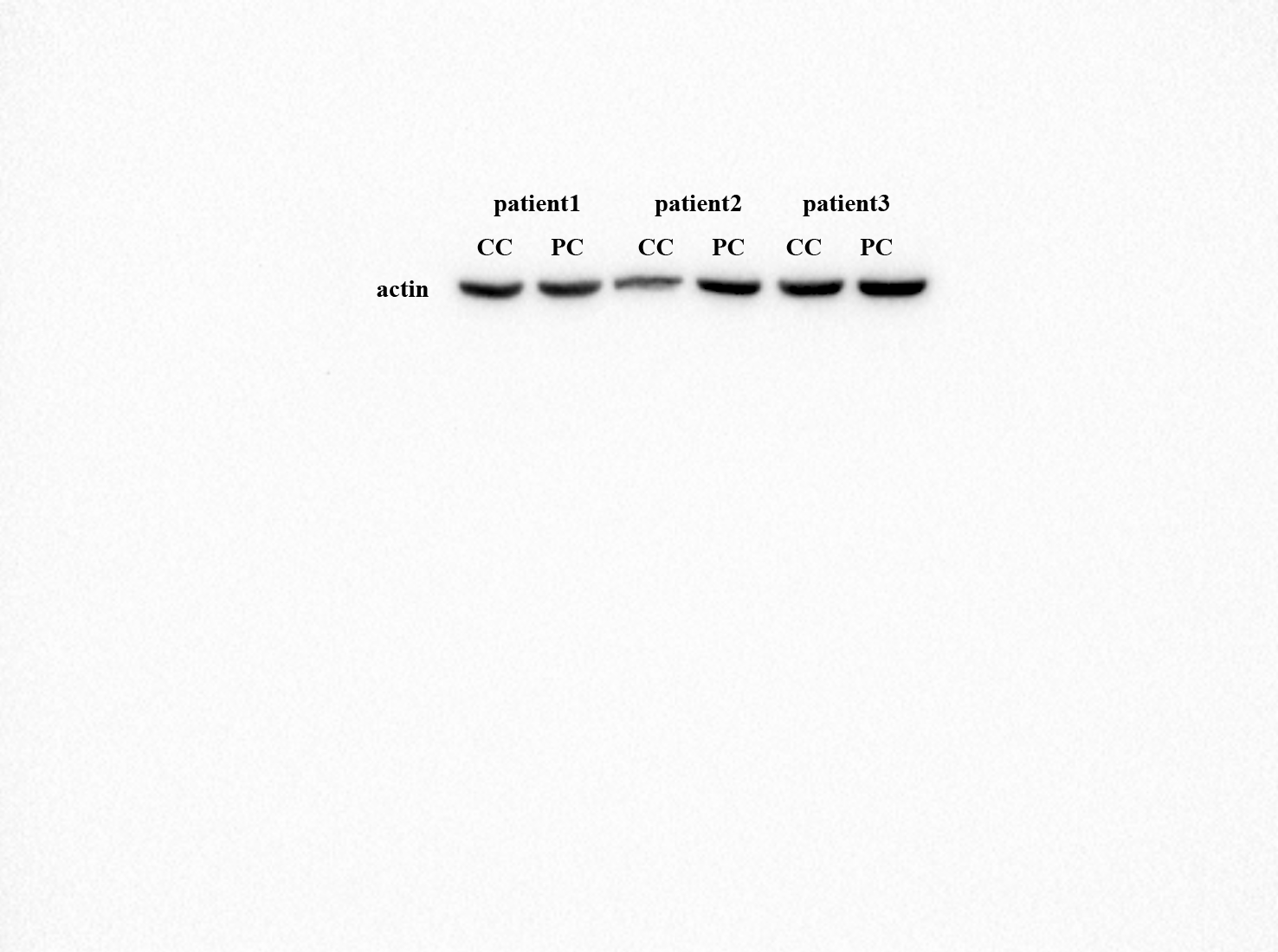
**

**Multiple exposure images were showed as follow:**

**actin**

**
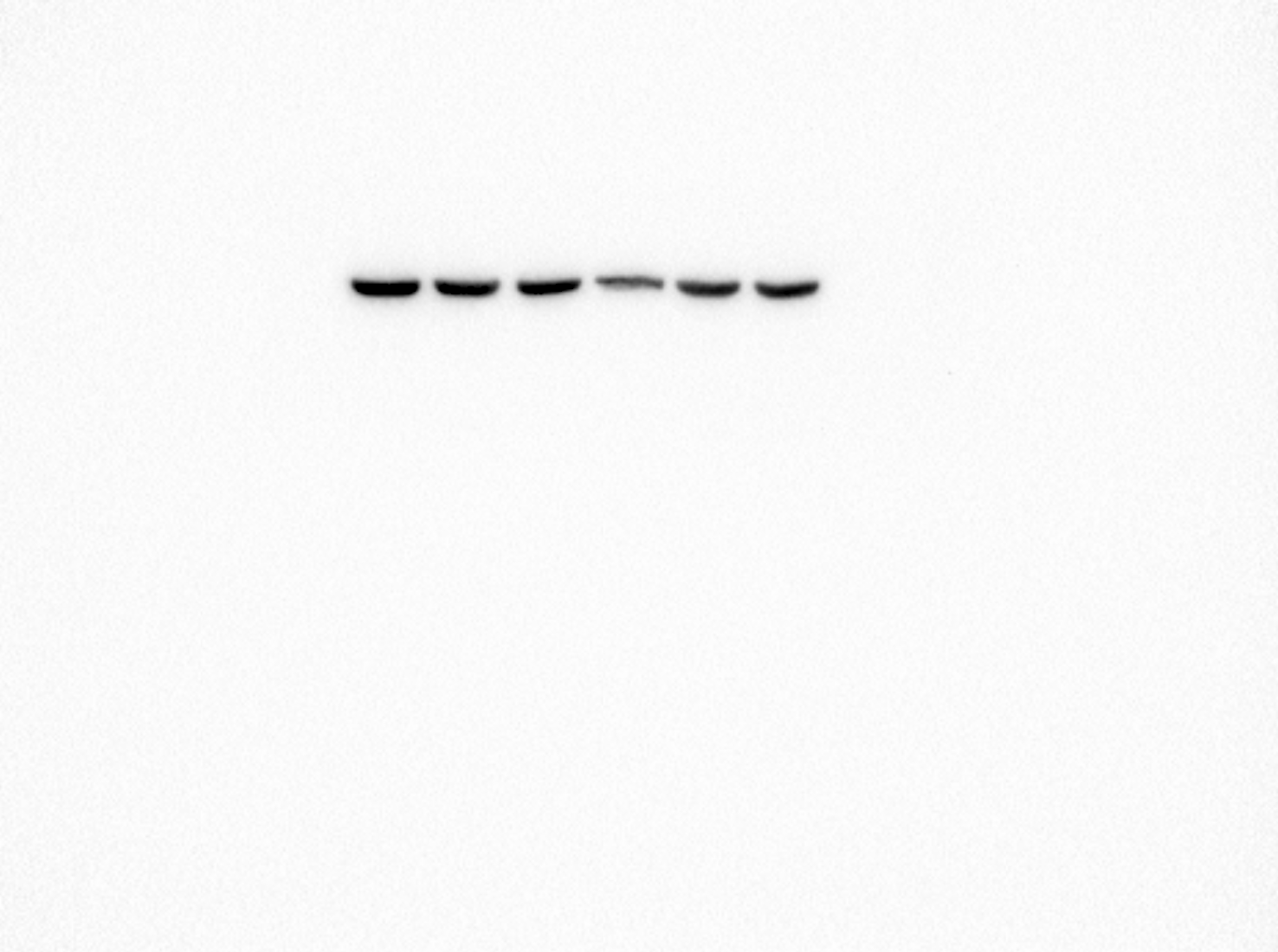

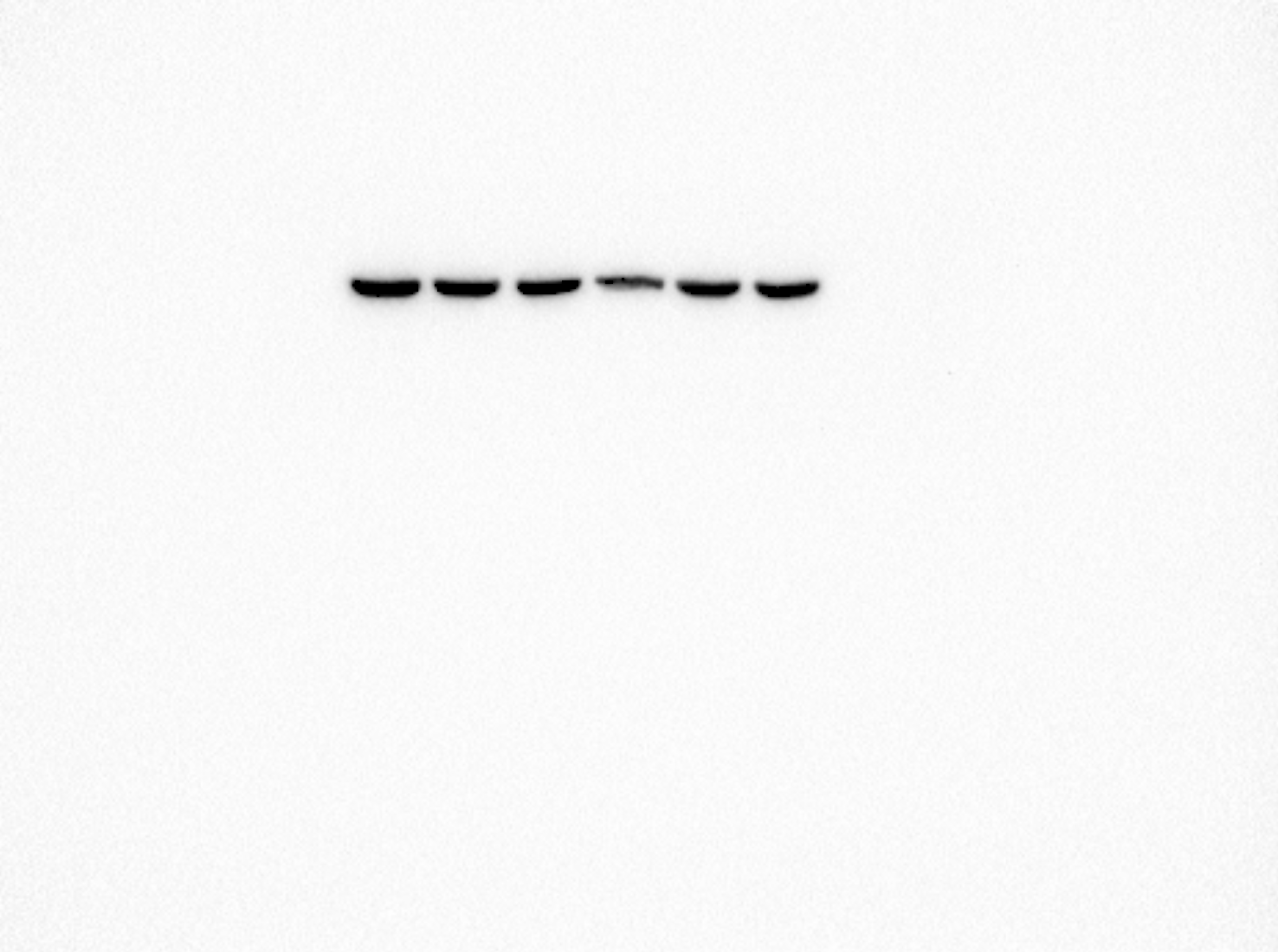
**

low exposure

medium exposure

**
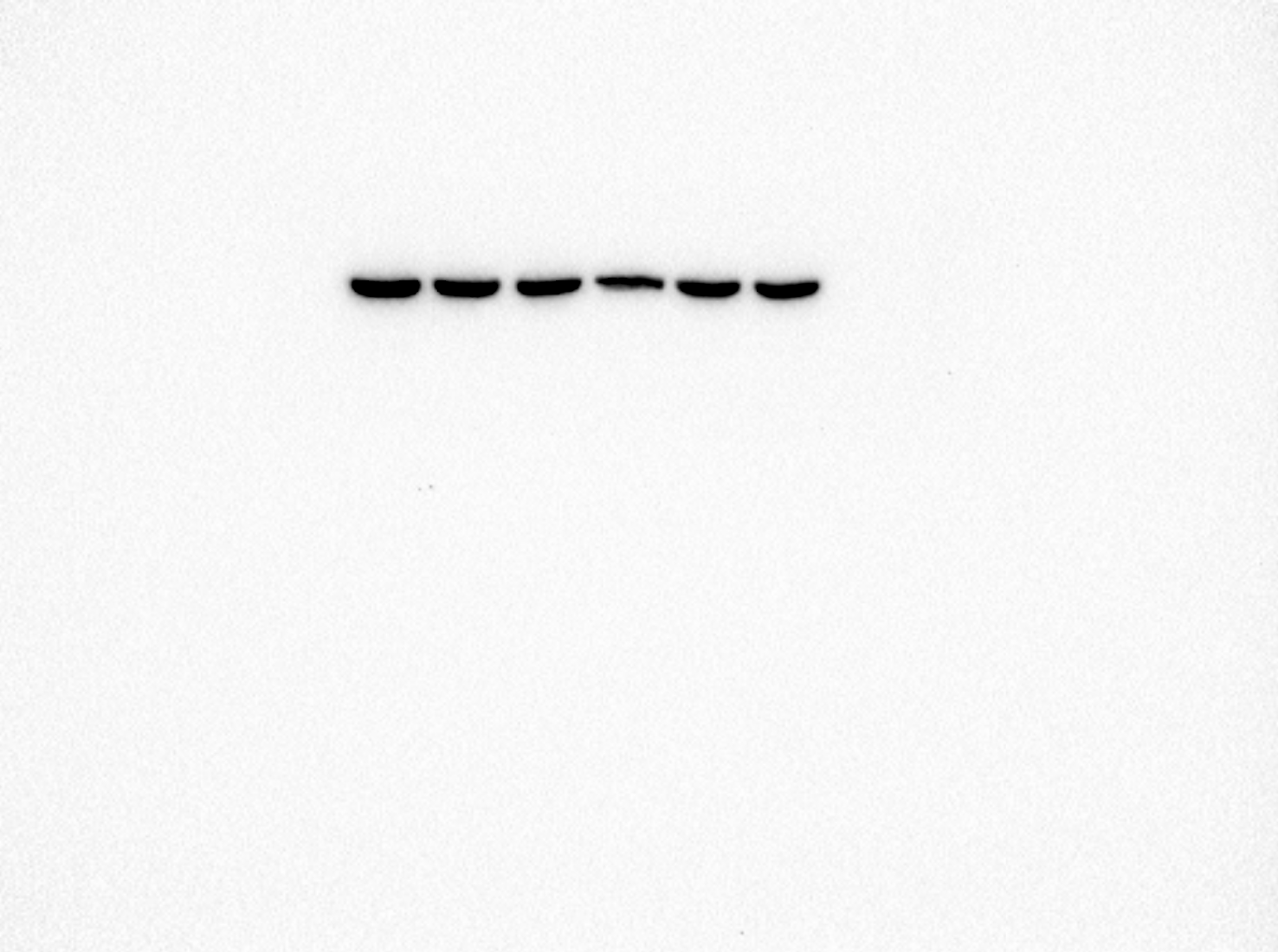
**

high exposure

**HSDL2**


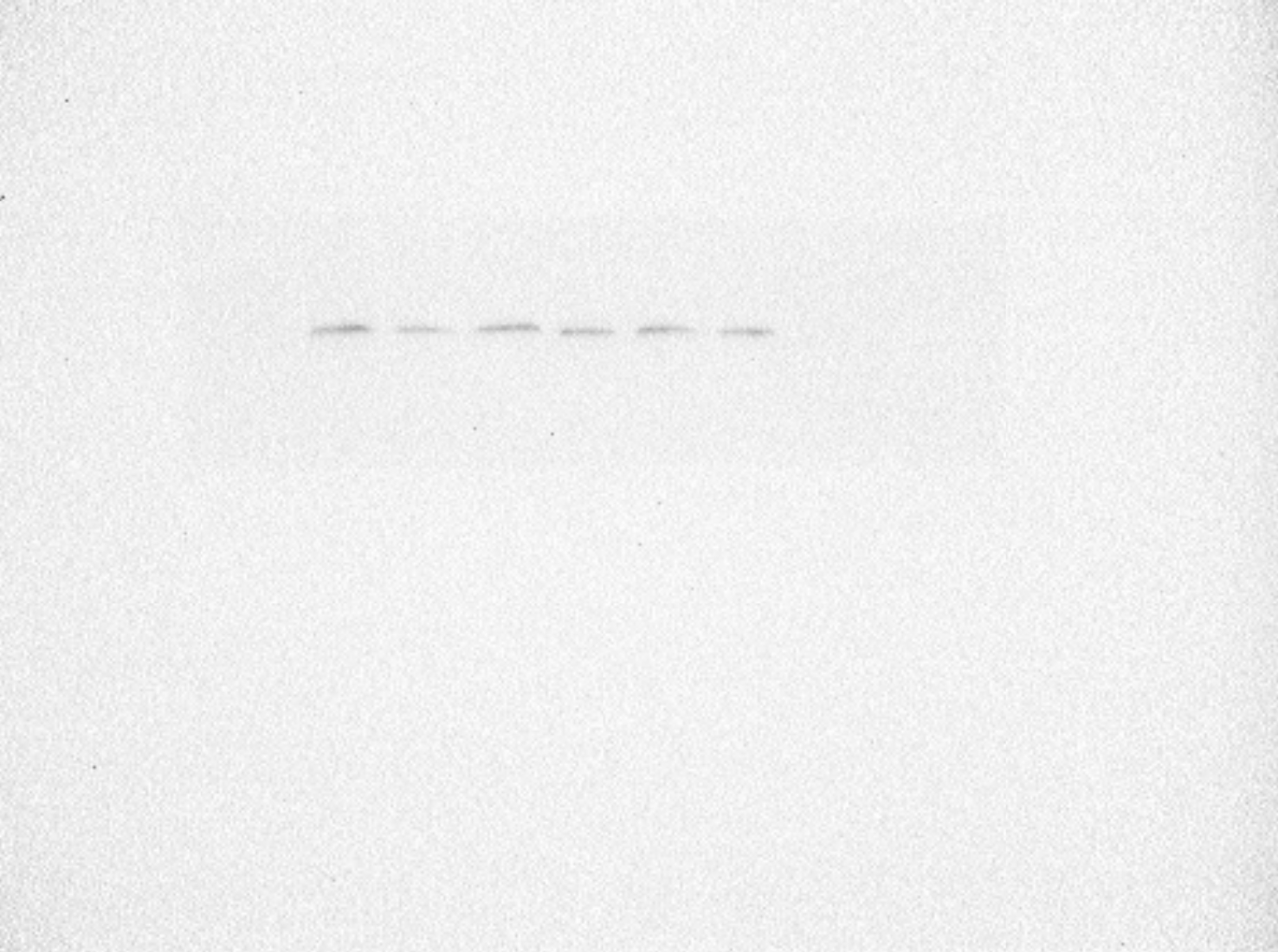

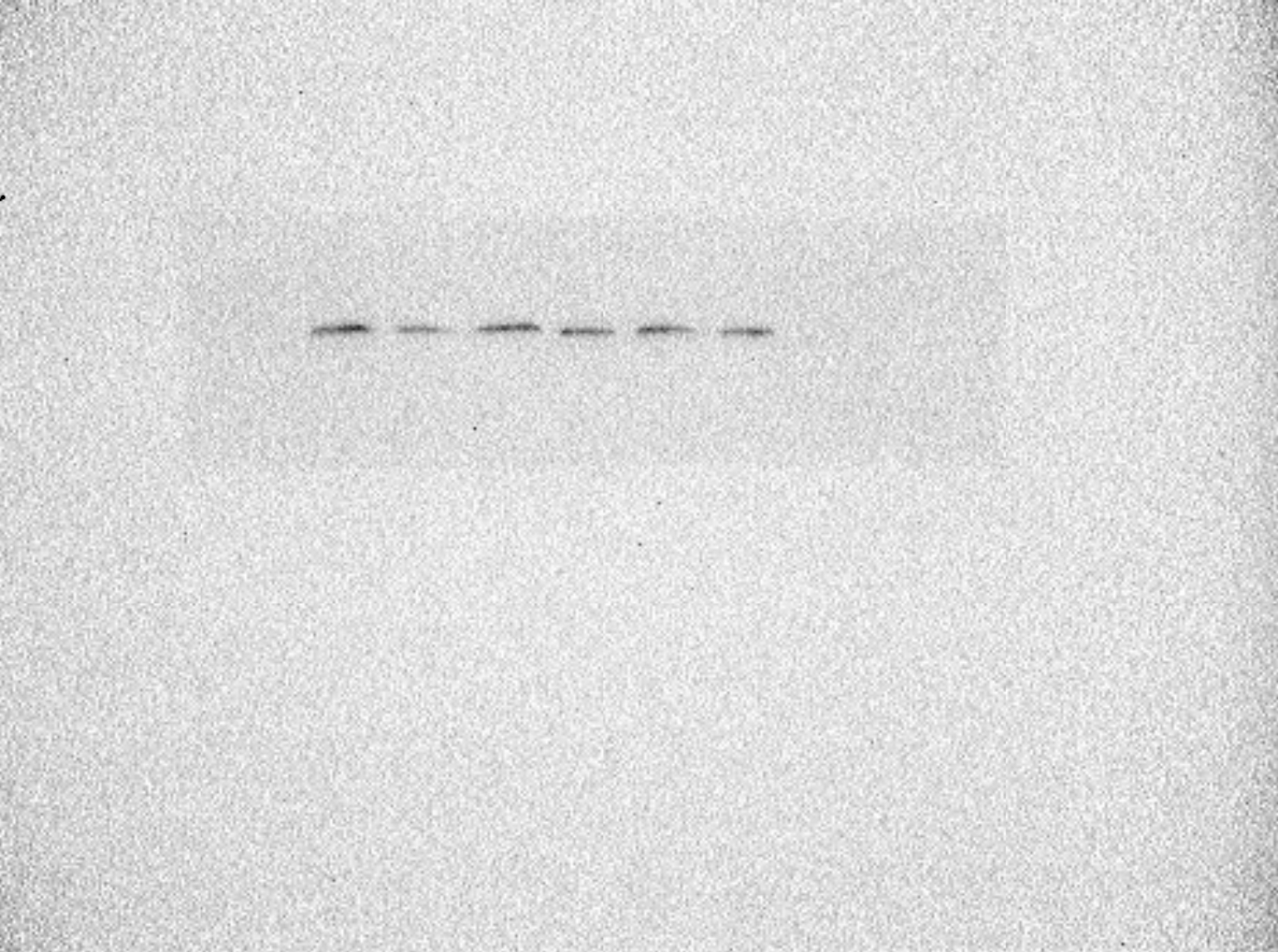


low exposure

medium exposure


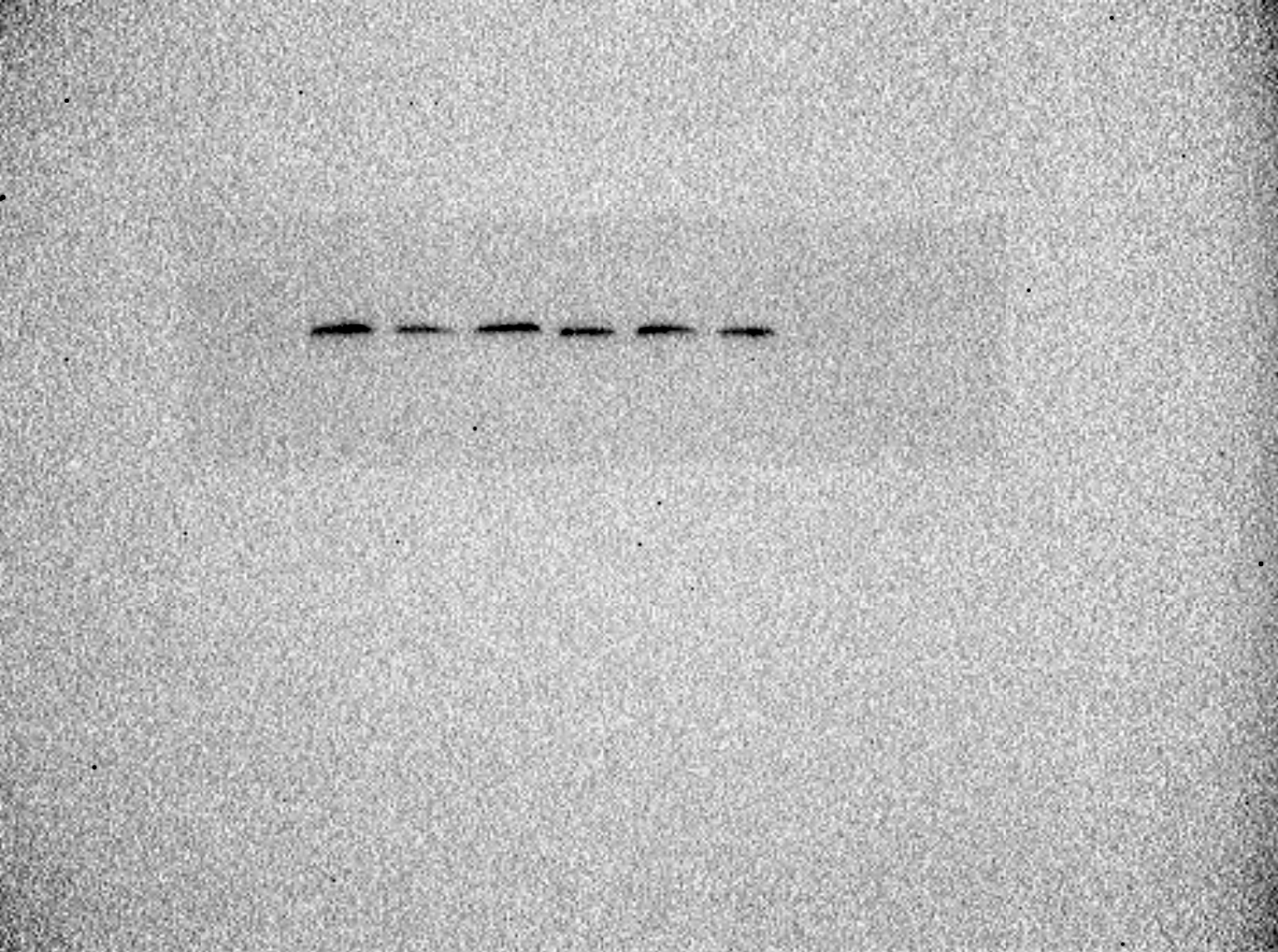


high exposure

**The original blots shown in the revised Figure 6A were listed as follow:**

**
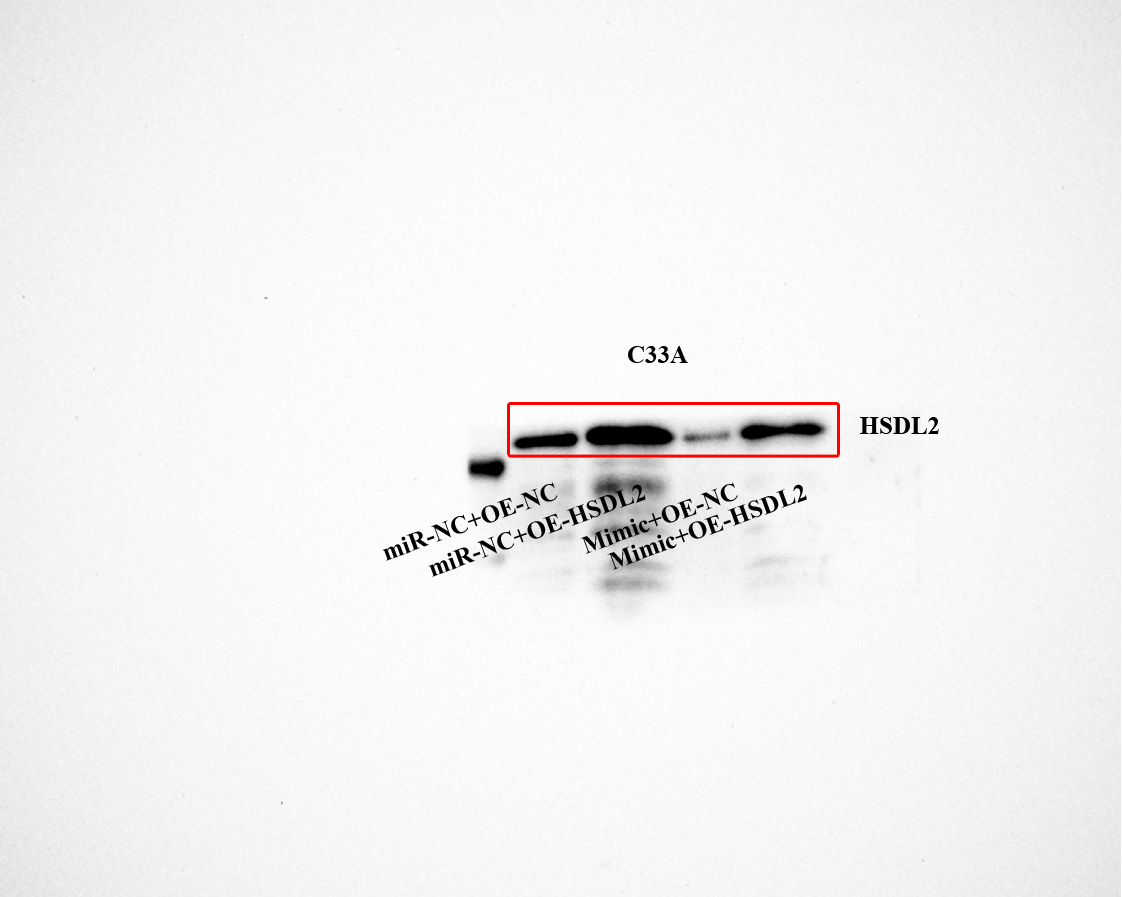

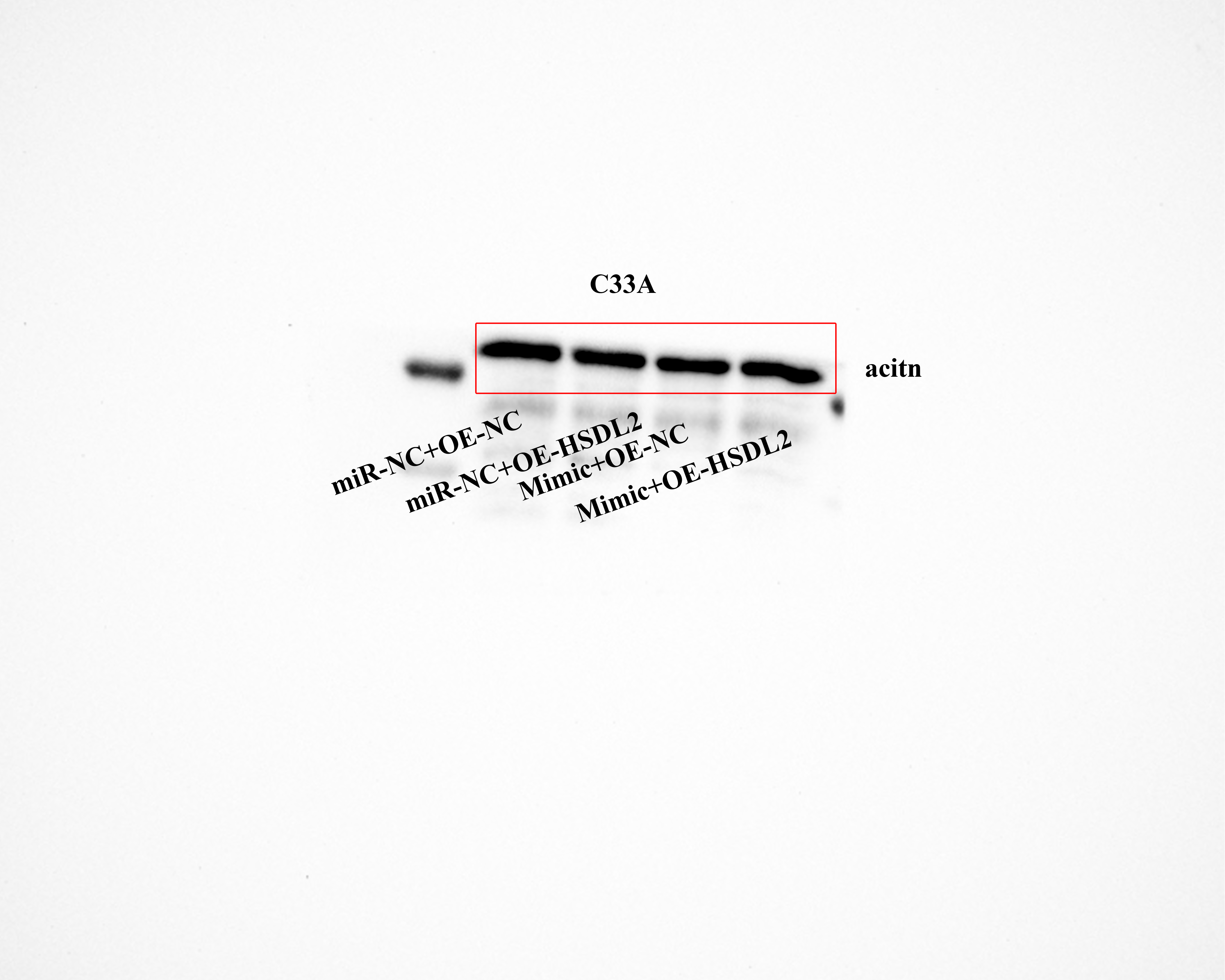
**

The red boxes show the cropped edge.

**The replicate images of blots in the C33A cells were listed as follow.**

**Replicate 1**

**
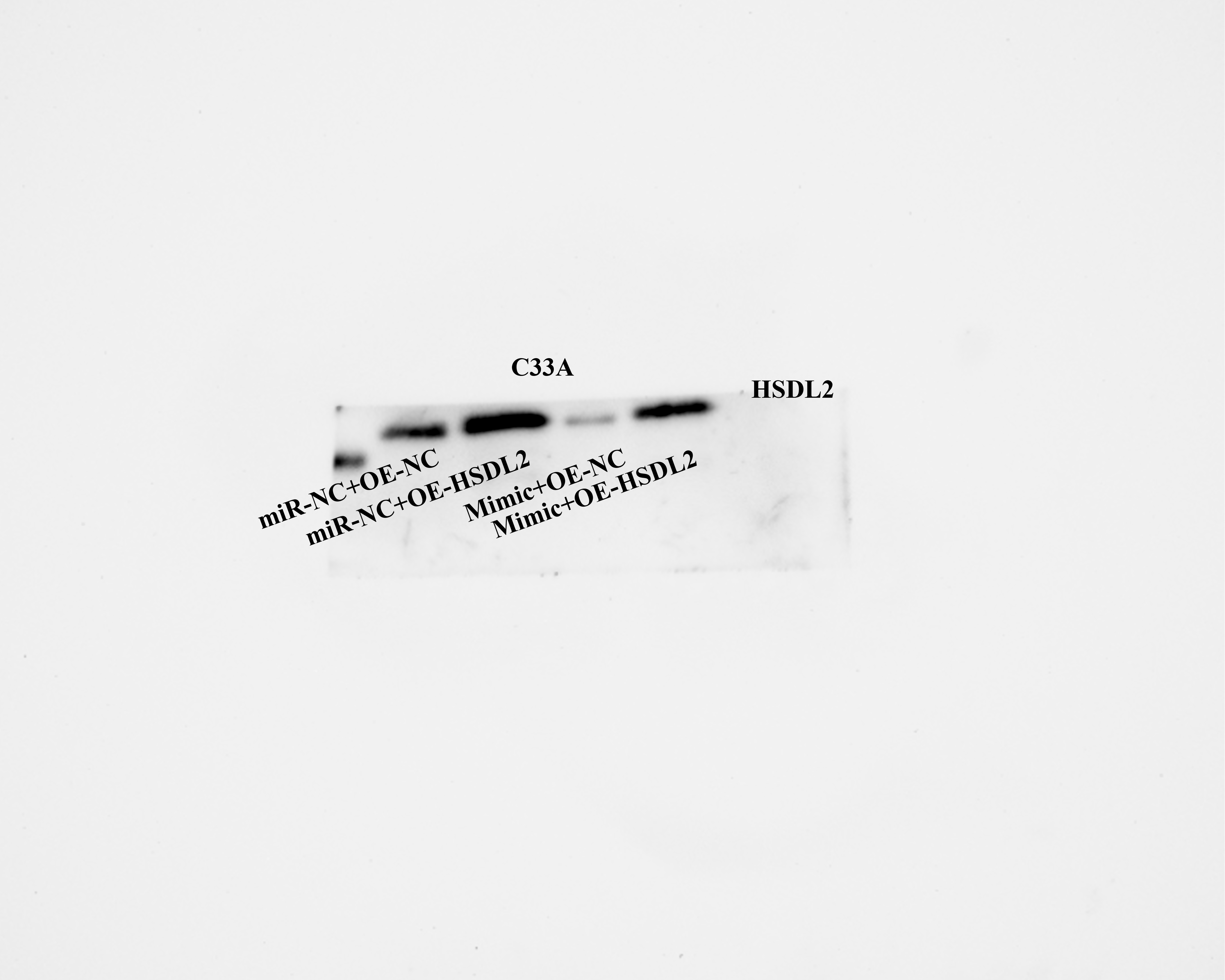

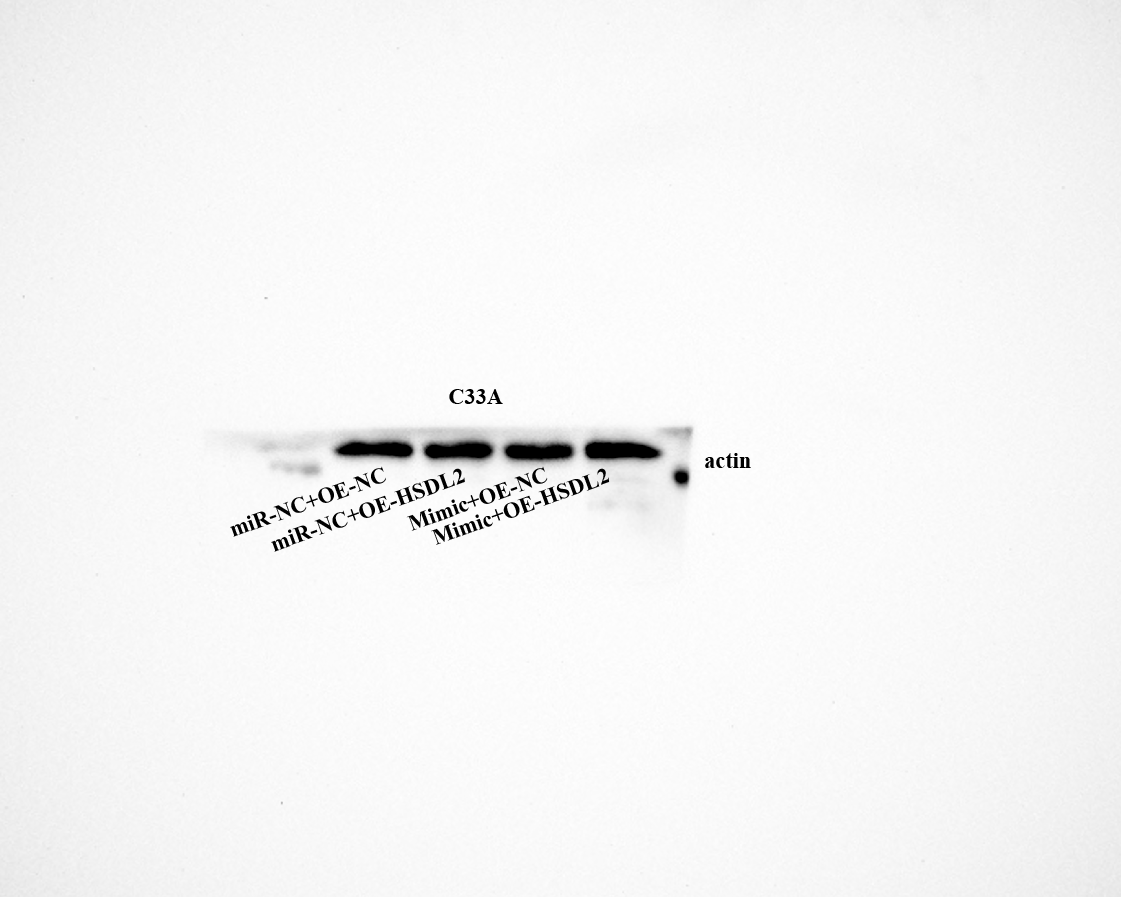
**

**Replicate 2:**


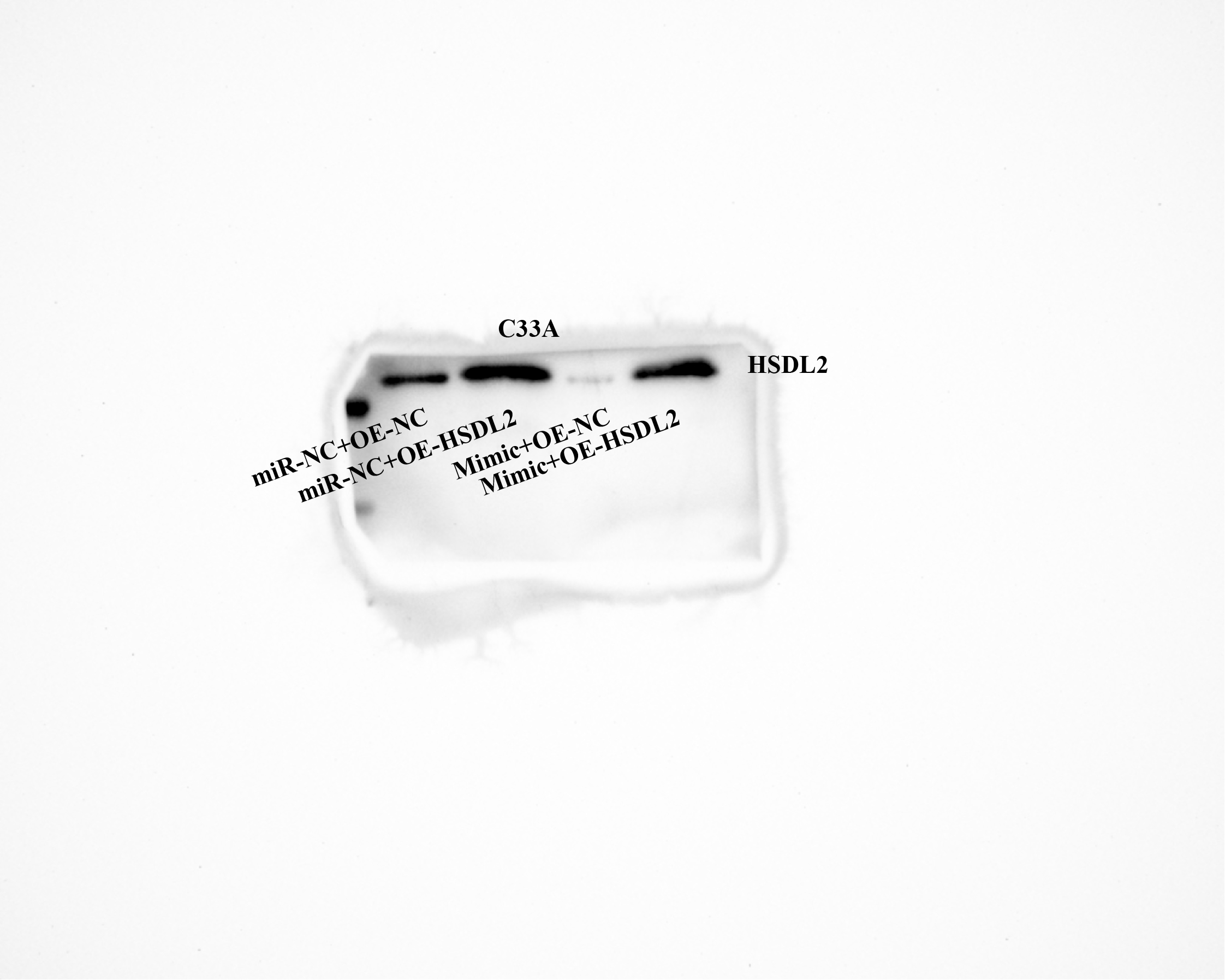

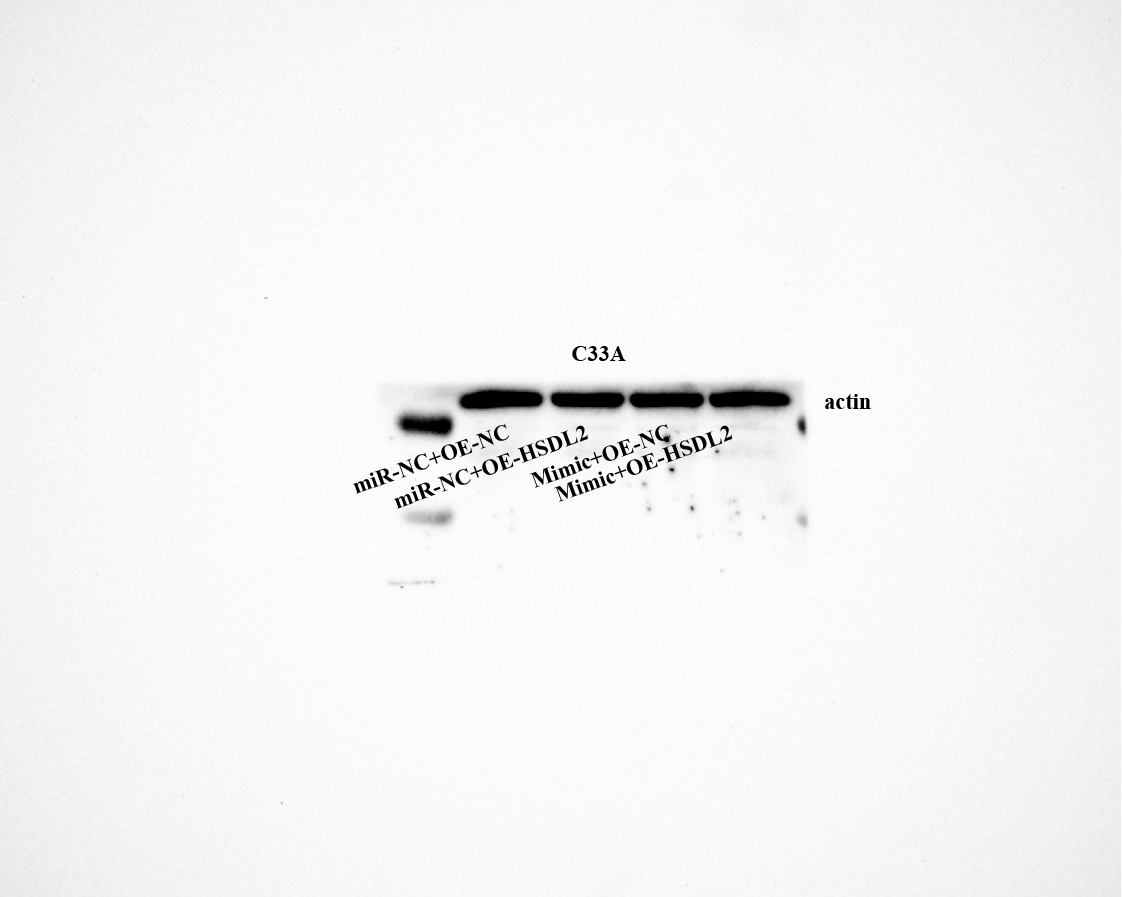


**The original blots shown in the revised Figure 6F were listed as follow:**


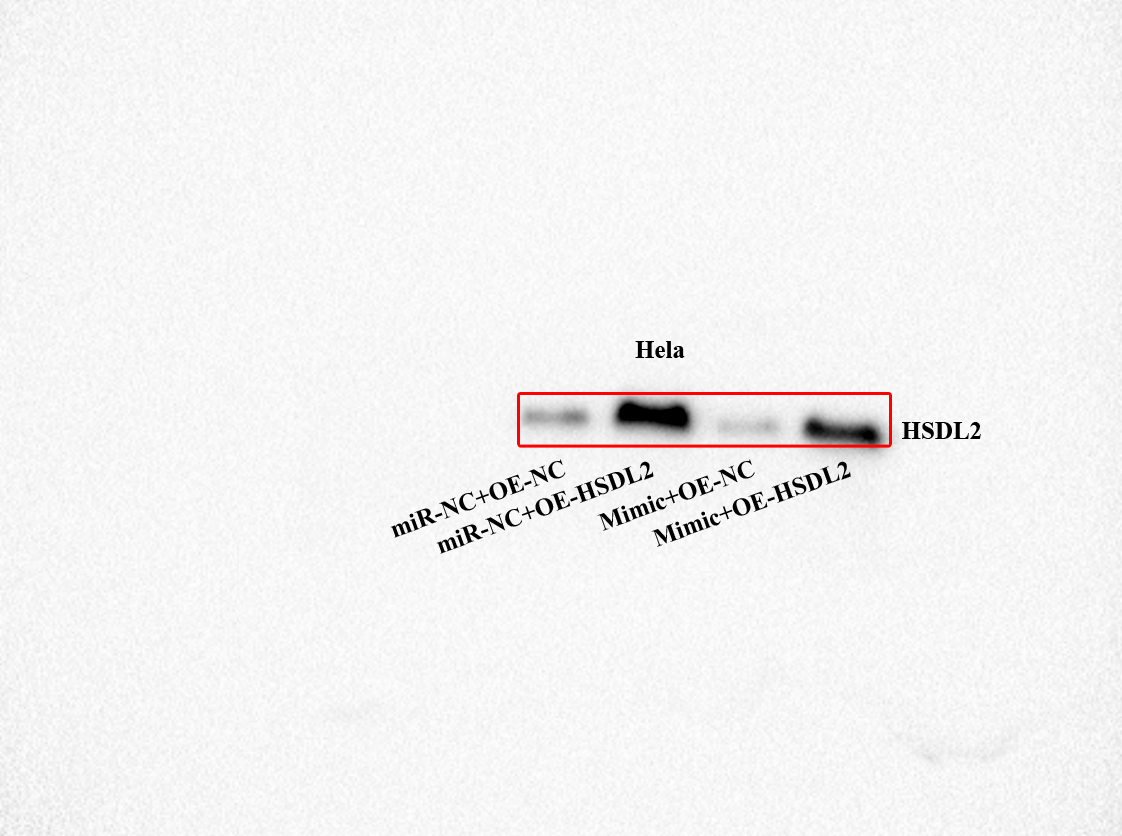

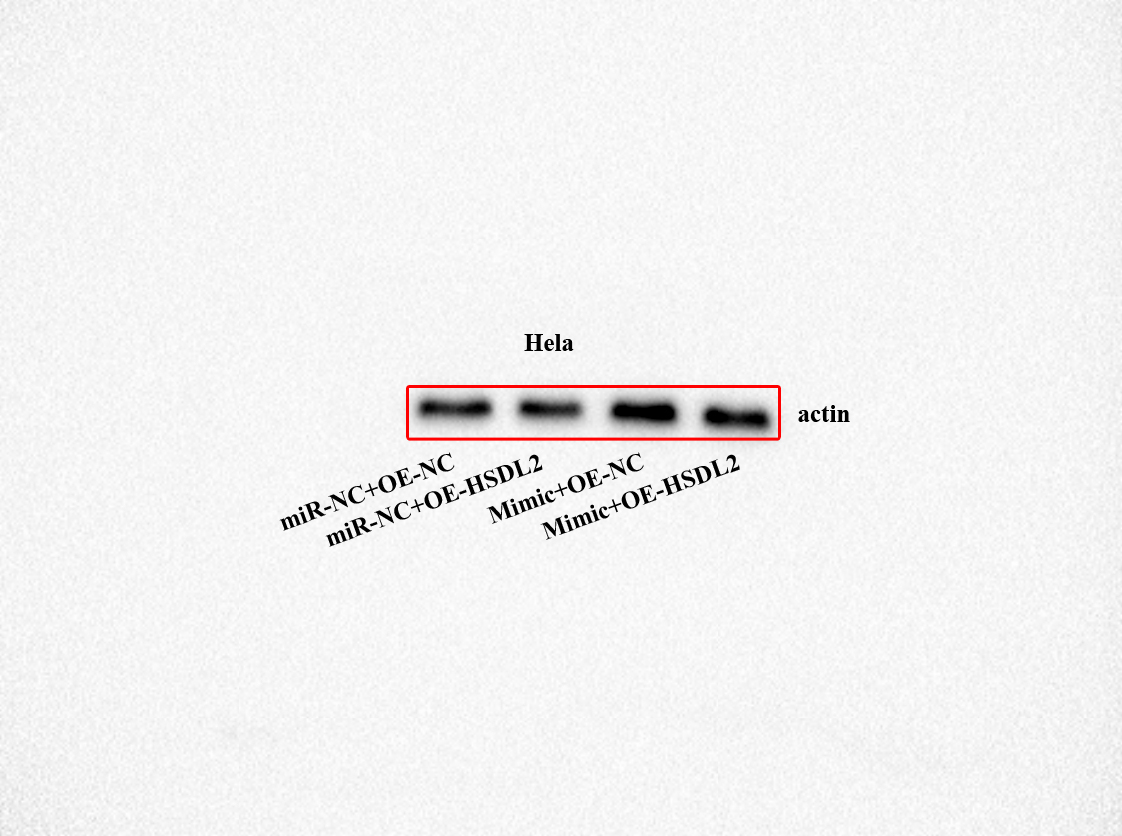


The red boxes show the cropped edge.

**The replicate images of blots in the Hela cells were listed as follow.**

**Replicate 1**


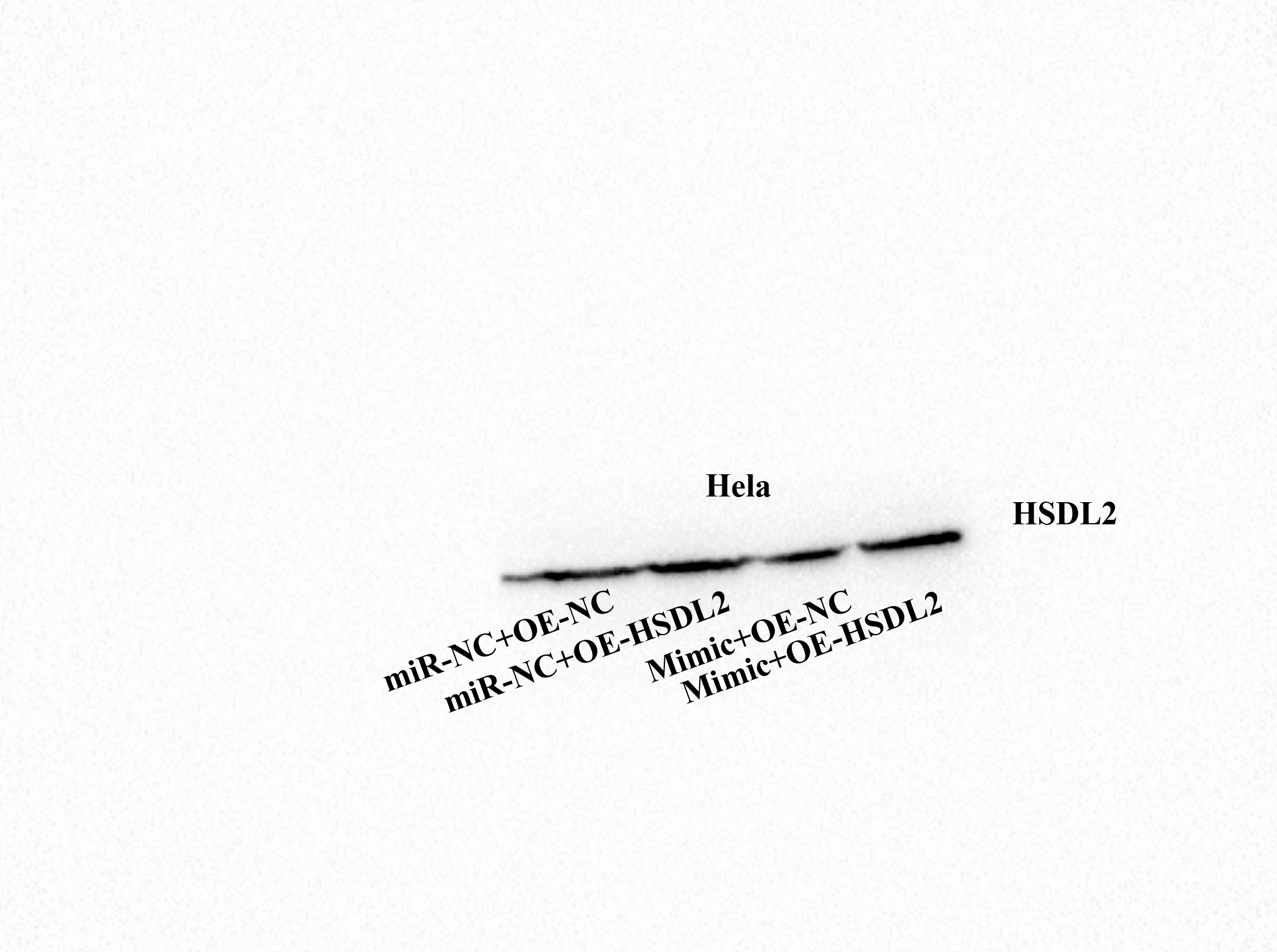

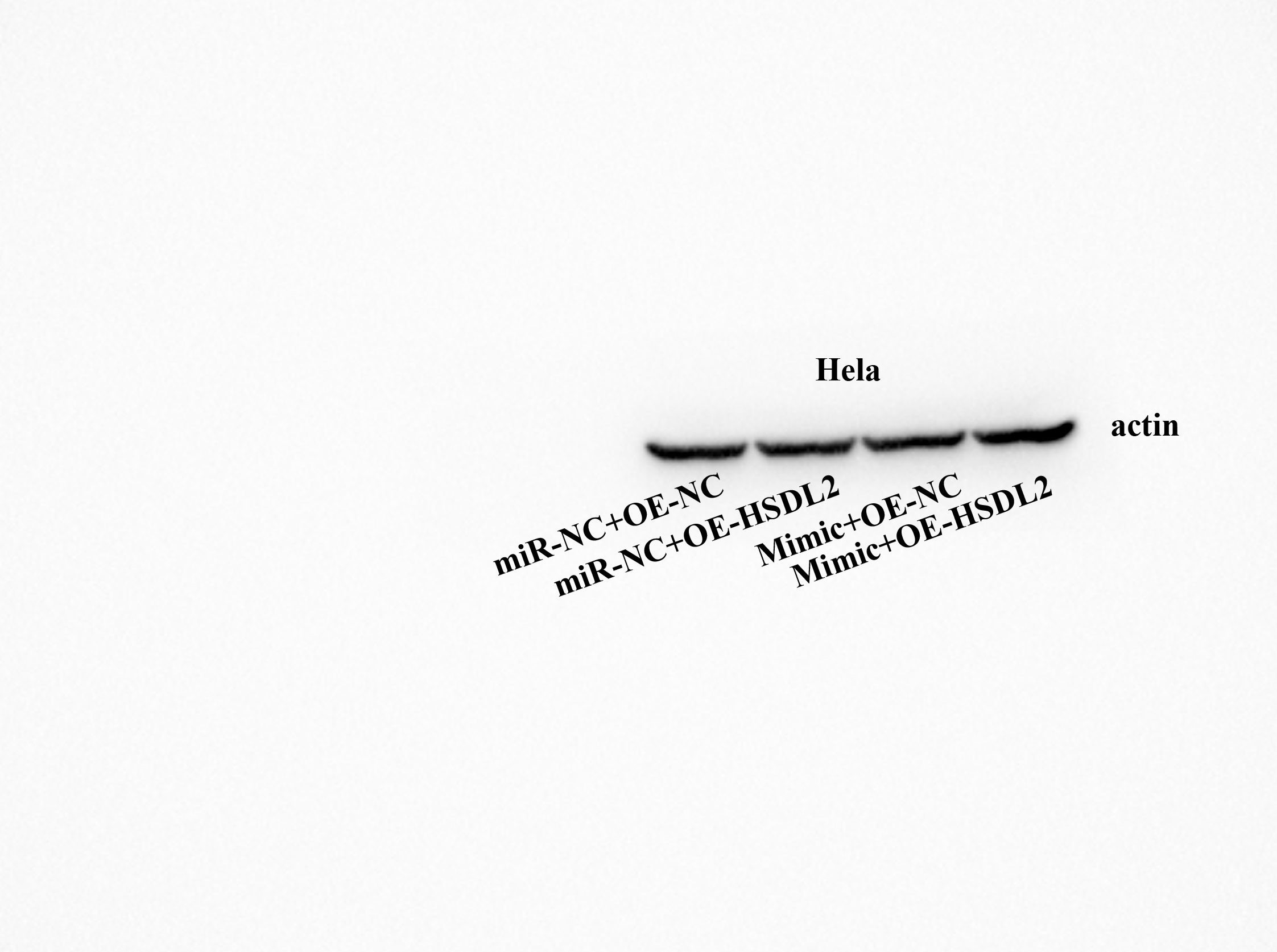


**Replicate 2**

**
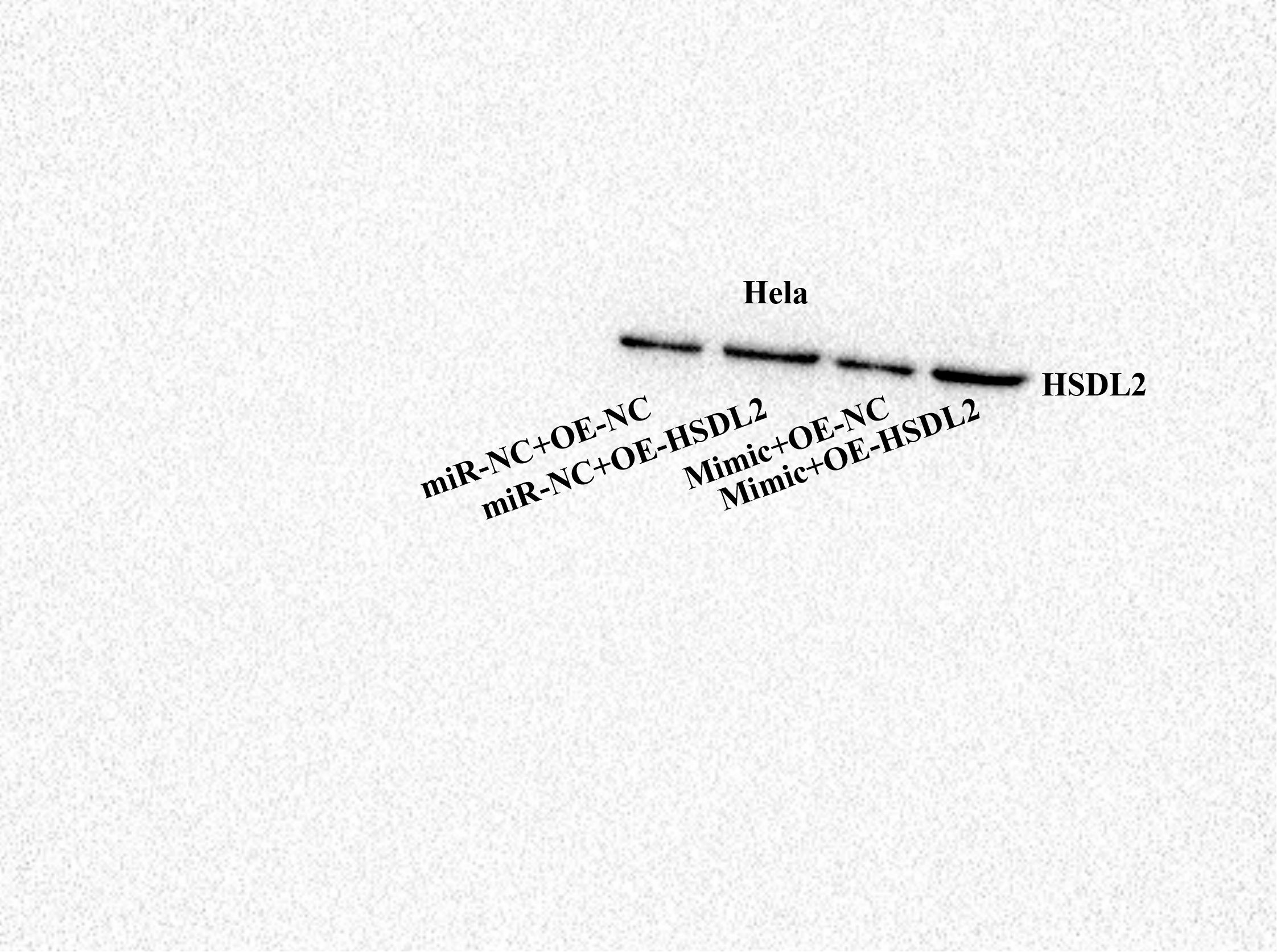

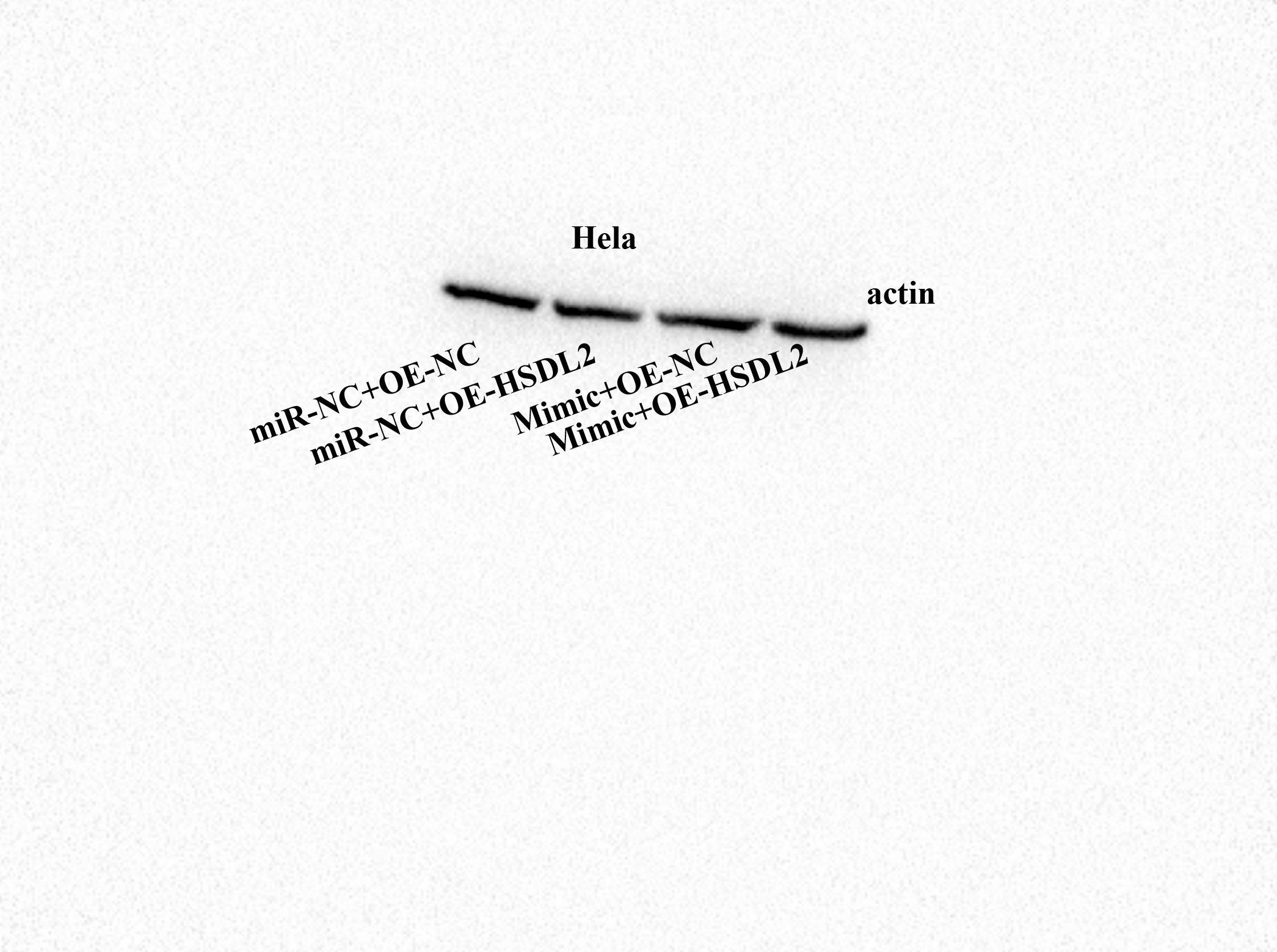
**
